# Clinical Benefits and Safety of Gemtuzumab Ozogamicin in Treating Acute Myeloid Leukemia in Various Subgroups: An Updated Systematic Review, Meta-Analysis, and Network Meta-Analysis

**DOI:** 10.3389/fimmu.2021.683595

**Published:** 2021-08-16

**Authors:** Qingyu Xu, Shujiao He, Li Yu

**Affiliations:** ^1^Department of Hematology and Oncology, International Cancer Center, Shenzhen Key Laboratory, Shenzhen University General Hospital, Shenzhen University Clinical Medical Academy, Shenzhen University Health Science Center, Shenzhen, China; ^2^Department of Hematology and Oncology, Medical Faculty Mannheim, Heidelberg University, Mannheim, Germany

**Keywords:** acute myeloid leukemia, gemtuzumab ozogamicin, response, survival, toxicity, meta-analysis, network meta-analysis

## Abstract

**Background:**

Previous trials demonstrated evidence involving the total effects of gemtuzumab ozogamicin (GO), an anti-CD33 humanized antibody, on treating acute myeloid leukemia (AML). In this updated systematic review, meta-analysis, and network meta-analysis (NMA), we aimed to comprehensively explore the clinical benefits and safety of GO in various subtypes of AML.

**Methods:**

PubMed, Embase, Cochrane, and Chinese databases were filtered to search randomized controlled trials (RCTs) and retrospective cohort studies that compared clinical efficiency and toxicity of GO with non-GO groups in AML. Random-effects models were used to calculate pooled effect sizes and 95% confidence intervals (CIs). Relative risk (RR) was used for estimating complete remission (CR), early death, and toxicity. Hazard risk (HR) was accomplished to evaluate survival.

**Results:**

Fifteen RCTs and 15 retrospective cohort studies were identified (GO: 4,768; Control: 6,466). GO tended to improve CR (RR 0.95, p = 0.084), followed by significantly improved survival (overall survival: HR 0.86, p = 0.003; event-free survival: HR 0.86, p = 0.015; relapse-free survival: HR 0.83, p = 0.001; cumulative incidence of relapse: HR 0.82, p < 0.001). GO benefits of CR and survival were evident in favorable- and intermediate-risk karyotypes (p ≤ 0.023). GO advantages were also associated with nucleophosmin 1 mutations (p ≤ 0.04), wild-type FMS-like tyrosine kinase 3 internal tandem duplication gene (p ≤ 0.03), age of <70 years (p < 0.05), *de novo* AML (p ≤ 0.017), and CD33(+) (p ≤ 0.021). Both adding GO into induction therapy (p ≤ 0.011) and a lower (<6 mg/m^2^) dose of GO (p ≤ 0.03) enhanced survival. Prognosis of combined regimens with GO was heterogeneous in both meta-analysis and NMA, with several binding strategies showing improved prognosis. Additionally, GO was related to increased risk of early death at a higher dose (≥6 mg/m^2^) (RR 2.01, p = 0.005), hepatic-related adverse effects (RR 1.29, p = 0.02), and a tendency of higher risk for hepatic veno-occlusive disease or sinusoidal obstruction syndrome (RR 1.56, p = 0.072).

**Conclusions:**

These data indicated therapeutic benefits and safety of GO in AML, especially in some subtypes, for which further head-to-head RCTs are warranted.

**Systematic Review Registration:**

[PROSPERO: https://www.crd.york.ac.uk/prospero/], identifier [CRD42020158540].

## Introduction

Acute myeloid leukemia (AML) is a heterogeneous hematological malignancy characterized by accumulated myeloid progenitor cells, leading to poor prognosis (1). High-risk factors such as age, cytogenetics, and genetics play a crucial role in predicting prognosis and influencing recommendations of therapies (2).

The conventional induction chemotherapy of AML combines anthracycline with cytarabine (Ara-C), such as daunorubicin plus Ara-C (DA) (3). However, these combined applications are associated with high toxicity (including thrombocytopenia, neutropenia, and anemia) and marginal rates of complete remission (CR) (53%–58%), particularly in elderly cohorts (4). Owing to the shortage of standard chemotherapy, immunotherapeutic strategies, such as antibodies against tumor antigens, might be promising in treating AML and have been proven to be highly effective in other hematological malignancies (5).

In AML, CD33 is frequently and specifically expressed on the surface of more than 90% of myelocytic and myelomonocytic precursor cells, such as blasts, rather than hematopoietic stem cells (HSCs) and outside of the hematological system. Gemtuzumab ozogamicin (GO) is a humanized antibody–drug conjugate composed of a monoclonal antibody targeting CD33, covalently linked to a semisynthetic derivative of calicheamicin. The GO binding to CD33 on AML blasts is followed by internalization of the GO–CD33 complex and toxin release intracellularly, leading to DNA damage and cell death (6). Due to targeting CD33, this complex is predicted to harbor higher specificity for harming AML cells without destroying normal HSCs and organs. Therefore, the expression of CD33 status might affect the therapeutic efficiency of GO. Initially approved by the US Food and Drug Administration (FDA) for treating relapsed AML, GO was subsequently voluntarily withdrawn due to excessive toxicity at higher doses (≥6 mg/m^2^) (7). However, later randomized clinical trials (RCTs), such as AML-15 (8), AML-16 (9), and ALFA-0701 (10), demonstrated that a lower dose of GO (3–5 mg/m^2^) plus DA improved survival. In addition to DA, GO added to other regimens, such as Ara-C monotherapy, FLAG (fludarabine, Ara-C, and granulocyte colony-stimulating factor), ADE (daunorubicin, Ara-C, and etoposide), and MICE (mitoxantrone, etoposide, and Ara-C) (8, 11–13), resulted in different treatment efficiencies. Except for CD33 status, doses of GO, and combined strategies, GO effects might also be affected by other clinical factors, including age stratifications, gender, mutations [such as mutated Nucleophosmin 1 (*NPM1*) and FMS-like tyrosine kinase-3 internal tandem duplication (*FLT3-ITD*)], *de novo* or secondary AML (sAML), cytogenetic risks, and treatment stages (9, 10, 14–16).

However, until now, no published study has comprehensively evaluated the therapeutic effectiveness of GO in all subgroups mentioned above. Therefore, we conducted this meta-analysis to evaluate GO in diverse patient populations to clarify the target cohort. We also performed a network meta-analysis (NMA) to compare GO effects between various combined therapies in RCT.

## Materials and Methods

This study was conducted according to the Preferred Reporting Items for Systematic reviews and Meta-Analyses (PRISMA) (17) (**Supplementary Table 1**), registered with PROSPERO (CRD42020158540).

### Search Strategy and Study Selection

A literature search was conducted by filtering databases of PubMed, Embase, Cochrane Library, Wanfang, and China National Knowledge Infrastructure since inception until August 31, 2020, following the search keywords containing “gemtuzumab ozogamicin”, “GO”, “Mylotarg”, “acute myeloid leukemia”, and “AML”. The included reports were (i) published in English or Chinese, (ii) restricted to retrospective cohort researches or RCT reporting therapeutic efficiency of GO in AML, and (iii) designed to include at least two arms comparing results between GO and non-GO groups regarding response information and survival outcomes. Studies were excluded if they (i) had unavailable or insufficient data; (ii) were editorials, letters, reviews, and case reports; (iii) had overlapped patient populations; or (iv) were single-arm studies.

Study selection was conducted in two steps. Initially, titles and abstracts of all potential literature were separately browsed and filtered by QX and SH based on inclusion and exclusion criteria. After removing duplicates, both reviewers screened potential reports again and decided their inclusion. Any discrepancy was discussed and, if necessary, settled through discussion or consultation with a third reviewer (LY). After selecting candidate studies, full articles were checked to identify final eligible studies.

### Assessment of Bias Risk and Study Quality

The methodologic quality of studies was independently estimated by two authors (QX and SH) through Newcastle–Ottawa Scale (NOS) (18) and Cochrane Risk of Bias Tool (19), which were used for cohort studies and RCTs, respectively. Any disparity was resolved by discussion. Publication bias was assessed with funnel plots as well as the Begg’s (20) and Egger’s tests (21) by Stata 15.1. A p-value <0.05 implied publication bias existence.

### Data Extraction

Clinical information was independently extracted from candidate studies by two authors (QX and SH). Any disagreement was settled by discussion or consultation with a third author (LY). The extracted data were composed of study characteristics (**Supplementary Table 2**) and prognostic information.

Prognostic endpoints included CR, overall survival (OS), event-free survival (EFS), relapse-free survival (RFS), and cumulative incidence of relapse (CIR), defined by revised International Working Group criteria (22), without required peripheral count recovery for CR. Relative risk (RR) and hazard ratio (HR) were used for estimating CR and survival outcomes, respectively. Data were preferentially extracted from multivariate analyses; otherwise, RR and HR were obtained from univariate analyses, Kaplan–Meier survival curves, or numeric reports as shown in the study from Tierney et al. (23).

### Statistical Analysis

The pooled RR and 95% confidence intervals (95% CIs) for CR were produced from the Mantel–Haenszel method, and the pooled HRs with 95% CI for OS, EFS, RFS, and CIR were calculated with the inverse variance method (24). All analyses were completed with Stata 15.1 software using random-effects models to obtain heterogeneity between studies. Pooled RR or HR <1.00 indicated better effect supporting GO treatment. It was considered statistically significant under the range of 95% CI without 1.00 or with a p-value <0.05. The χ^2^-based Q statistic estimated the heterogeneity among studies. Low, moderate, substantial, and considerable heterogeneity indicated *I* ^2^ < 30%, 30%–50%, 50%–75%, and >75%, respectively (25). A p-value ≥0.10 meant no or slight heterogeneity, whereas p-value <0.10 showed significant heterogeneity, which was settled by sensitivity and subgroup analyses to identify the source.

Bayesian NMA was done with R 4.0.2 software by means of a random model *via* packages of “gemtc” and “rjags” in RCT. We calculated HRs or RRs regarding non-GO group as the baseline to act as the effect measure, displayed in forest plots, where RR and HR with 95% credible intervals (95% Crls) were utilized to explain the extent of effects in CR and survival, respectively. To estimate relative HR and RR, a Markov Chain Monte Carlo simulation was finished with 10,000 adaptations and 100,000 iterations of each of the three automatically generated Markov chains. After completing all simulations, NMA determined the probability that each therapy would be best by calculating the probability of simulations in which a certain treatment ranked best. For each iteration, regimens were ranked based on the assessed log HR or log RR. The results from Bayesian NMA were compared with data from pairwise meta-analyses to estimate inconsistency using the node splitting method (26). If no closed-loop was present in the network evidence plot, inconsistency analysis could not be executed.

All analyses were based on published data. No ethical approval and patient consent were required.

## Results

### Studies Characteristics

A total of 1,170 references were retrieved from searching databases, 214 duplicates of which were initially removed. Of the remaining 956 records, 783 studies were excluded, since they did not fulfill the predefined inclusion criteria. The remaining 173 reports were retrieved for detailed full-text estimation. Finally, 30 studies were comprehensively analyzed. The **Supplementary Figure 1** illustrated the flow diagram of the study selection. Fifteen RCTs and 15 retrospective cohort studies were eventually contained in this study. Quality assessment of RCTs was shown in **Supplementary Figure 2**. For survival endpoints, we thought that bias was unlikely since death and relapse were not susceptible to patients, physicians, or outcome assessor bias. The details of NOS score for retrospective cohort studies were listed in **Supplementary Table 3**.

In total, 11,234 patients were contained, comprising 11,105 AML patients (8–15, 27–48) and 129 high-risk myelodysplastic syndrome (MDS) cases (9, 48). All studies compared therapeutic effects between GO (N = 4,768) and non-GO (N = 6,466) arms. Here, 796 patients had low-/intermediate-risk cytogenetics (GO: 398/796); 853 in favorable risk (GO: 375/853); 2,650 in intermediate risk (GO: 1,316/2,650); and 1,144 in inferior risk (GO: 540/1,144). The GO doses varied among studies (3–5 mg/m^2^: 3,098 cases; ≥6 mg/m^2^: 1,530 cases). GO was administered in induction regimen (3,649/8,082), consolidation strategy (793/1,852), and post-consolidation treatment (138/280).

### Pooled Prognosis of Gemtuzumab Ozogamicin

All analyses involved in CR, OS, EFS, RFS, and CIR were summarized into **Supplementary Tables 4–8**, respectively, including results before and after sensitivity analyses as well as subgroup analyses.

**Figure 1:**
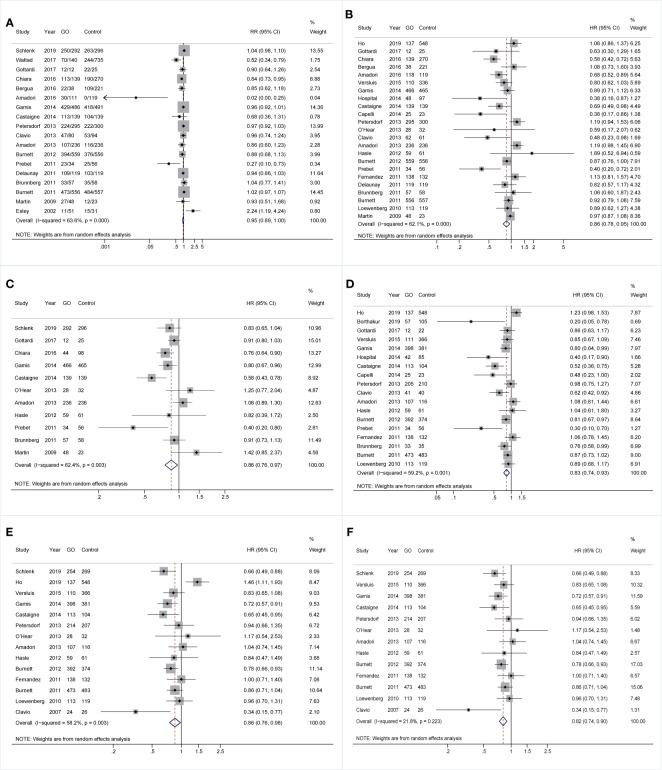
Pooled prognosis between GO and non-GO groups with AML. **(A)** Pooled CR. **(B)** Pooled OS. **(C)** Pooled EFS. **(D)** Pooled RFS. **(E)** Pooled CIR. **(F)** Pooled CIR after sensitivity analyses. The diamonds represent the overall summary RR and HR estimates with 95% CI. GO, gemtuzumab ozogamicin; AML, acute myeloid leukemia; CR, complete remission; OS, overall survival; EFS, event-free survival; RFS, relapse-free survival; CIR, cumulative incidence of relapse; RR, relative risk; HR, hazard ratio; 95% CI, 95% confidence interval.

Comparable pooled CRs were achieved between GO (73.32%, 2,487/3,392) and non-GO groups (64.52%, 2,791/4,326) (RR 0.95; 95% CI 0.89–1.00, p = 0.084) with substantial heterogeneity (**Figure 1A**). However, benefits of GO were observed in all survival outcomes (OS: HR 0.86; 95% CI 0.78–0.95, p = 0.003; EFS: HR 0.86; 95% CI 0.76–0.97, p = 0.015; RFS: HR 0.83; 95% CI 0.74–0.93, p = 0.001; CIR: HR 0.86; 95% CI 0.76–0.98, p = 0.020; **Figures 1B–E**), accompanied by substantial heterogeneity. Sensitivity analyses demonstrated that only the heterogeneity of pooled CIR was overcome by removing the study from Ho et al. (27), showing pooled HR of 0.82 (95% CI 0.74–0.90, p = 0.000; **Figure 1F**).

### Subgroup Analyses Regarding Karyotypes and Mutations

**Figure 2:**
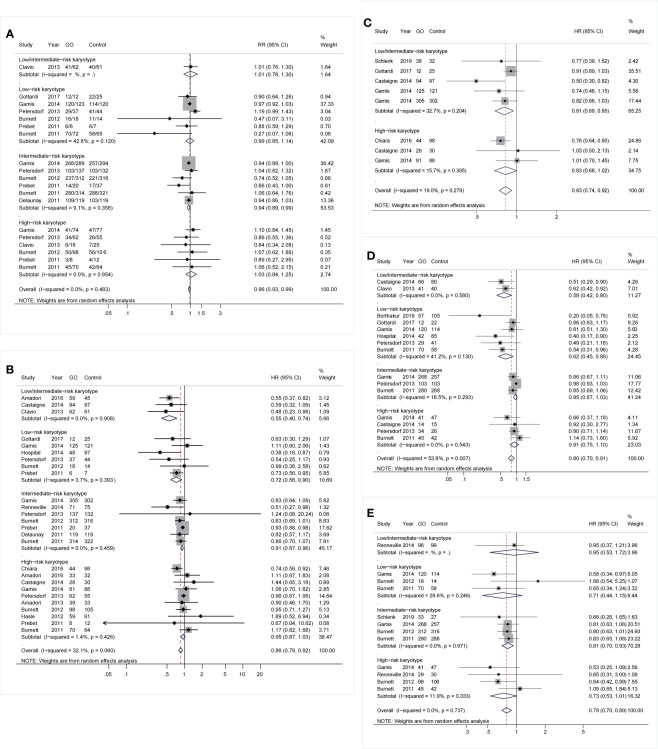
Prognostic subgroup analyses regarding karyotype stratifications of AML. **(A)** CR. **(B)** OS. **(C)** EFS. **(D)** RFS. **(E)** CIR. The diamonds represent the overall summary RR and HR estimates with 95% CI. GO, gemtuzumab ozogamicin; AML, acute myeloid leukemia; CR, complete remission; OS, overall survival; EFS, event-free survival; RFS, relapse-free survival; CIR, cumulative incidence of relapse; RR, relative risk; HR, hazard ratio; 95% CI, 95% confidence interval.

Based on subgroup analyses of karyotypes, patients benefited from GO at low- and intermediate-risk karyotypes instead of adverse-risk cytogenetics (**Figure 2**). CR was slightly improved in intermediate-risk karyotype (RR 0.94; 95% CI 0.89–0.99, p = 0.023; **Figure 2A**) with low heterogeneity. Besides, GO consistently favored better OS in intermediate-risk cytogenetics (HR 0.91; 95% CI 0.87–0.96, p < 0.001) as well as in low-/intermediate-risk (HR 0.55; 95% CI 0.40–0.74, p < 0.001) and favorable-risk karyotypes (HR 0.72; 95% CI 0.58–0.90, p = 0.003) after sensitivity analyses (**Figure 2B**). EFS, RFS, and CIR of GO were consistently improved in low-/intermediate-risk (HR 0.81; 95% CI 0.69–0.95, p = 0.010; **Figure 2C**), low-risk (HR 0.62; 95% CI 0.45–0.86, p = 0.004; **Figure 2D**), and intermediate-risk (HR 0.81; 95% CI 0.70–0.93, p = 0.004; **Figure 2E**) cytogenetics, respectively.

**Figure 3:**
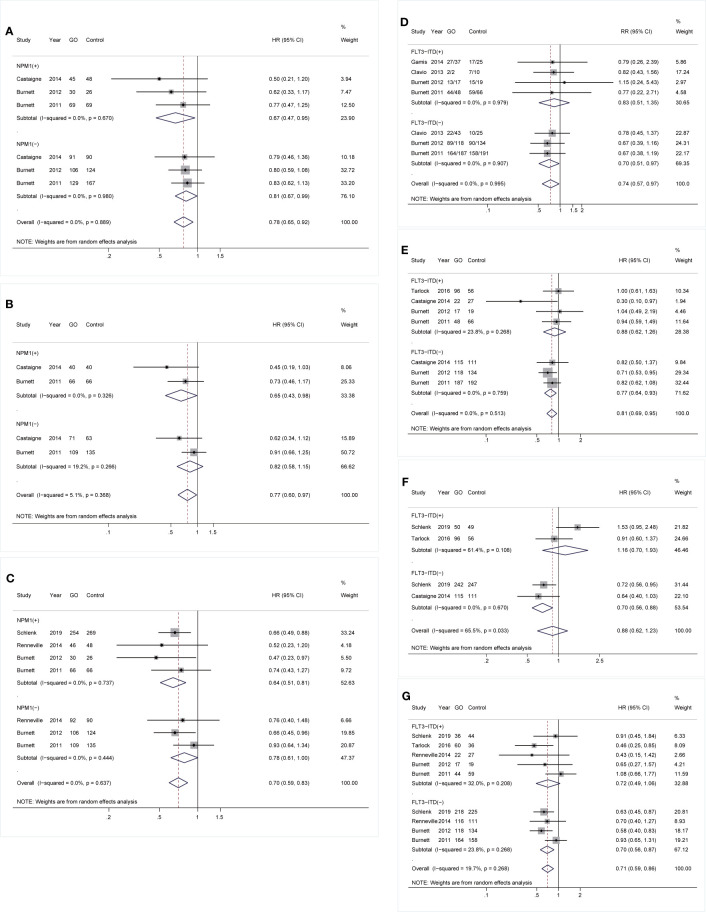
Prognostic subgroup analyses regarding NPM1 and FLT3-ITD mutations. **(A)** OS subgrouped by NPM1 mutational status. **(B)** RFS subgrouped by NPM1 mutational status. **(C)** CIR subgrouped by NPM1 mutational status. **(D)** CR subgrouped by FLT3-ITD mutational status. **(E)** OS subgrouped by FLT3-ITD mutational status. **(F)** EFS subgrouped by FLT3-ITD mutational status. **(G)** CIR subgrouped by FLT3-ITD mutational status. The diamonds represent the overall summary RR and HR estimates with 95% CI. GO, gemtuzumab ozogamicin; CR, complete remission; OS, overall survival; EFS, event-free survival; RFS, relapse-free survival; CIR, cumulative incidence of relapse; RR, relative risk; HR, hazard ratio; 95% CI, 95% confidence interval; NPM1, nucleophosmin 1; FLT3-ITD, FMS-like tyrosine kinase 3 internal tandem duplication.

As for mutations, GO was linked to consistently better OS and CIR regardless of *NPM1* mutation [OS: *NPM1(+)*: HR 0.67; 95% CI 0.47–0.95, p = 0.026; *NPM1(-)*: HR 0.81; 95% CI 0.67–0.99, p = 0.034, **Figure 3A**; CIR: *NPM1(+)*: HR 0.64; 95% CI 0.51–0.81, p < 0.001; *NPM1(-)*: HR 0.78; 95% CI 0.61–1.00, p = 0.049, **Figure 3C**]. However, RFS was only consistently increased in GO of *NPM1*(+) cohort (HR 0.65; 95% CI 0.43–0.98, p = 0.040; **Figure 3B**). Besides, CR was consistently enhanced in GO of *FLT3-ITD*(-) subjects (RR 0.70; 95% CI 0.51–0.97, p = 0.030; **Figure 3D**), followed by better OS (HR 0.77; 95% CI 0.64–0.93, p = 0.006; **Figure 3E**), EFS (HR 0.70; 95% CI 0.56–0.88, p = 0.002; **Figure 3F**), and CIR (HR 0.70; 95% CI 0.56–0.87, p = 0.002; **Figure 3G**).

### Subgroup Analyses Regarding Age, Genders, Acute Myeloid Leukemia Types, and CD33 Status

**Figure 4:**
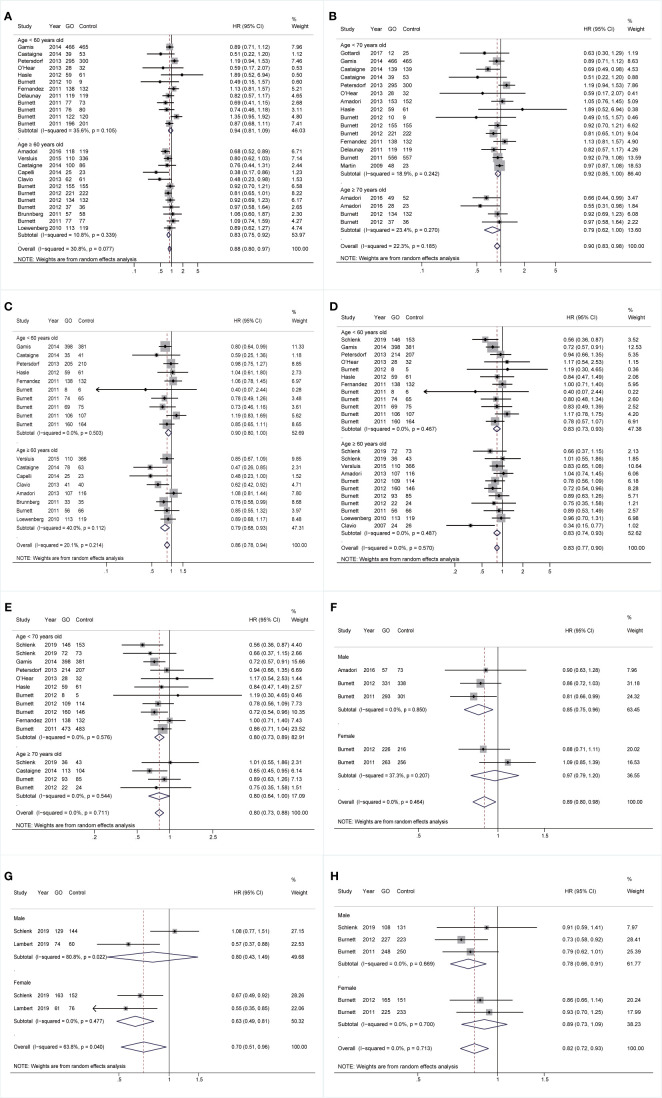
Prognostic subgroup analyses regarding age stratifications and gender. **(A)** OS subgrouped by age (60 years old). **(B)** OS subgrouped by age (70 years old). **(C)** RFS subgrouped by age (60 years old). **(D)** CIR subgrouped by age (60 years old). **(E)** CIR subgrouped by age (70 years old). **(F)** OS subgrouped by gender. **(G)** EFS subgrouped by gender. **(H)** CIR subgrouped by gender. The diamonds represent the overall summary HR estimates with 95% CI. GO, gemtuzumab ozogamicin; OS, overall survival; EFS, event-free survival; RFS, relapse-free survival; CIR, cumulative incidence of relapse; HR, hazard ratio; 95% CI, 95% confidence interval.

With respect to age, for cohorts aged ≥60 years, GO supported better OS (HR 0.83; 95% CI 0.75–0.92, p < 0.001; **Figure 4A**) after sensitivity analyses and consistently achieved better RFS (HR 0.79; 95% CI 0.68–0.93, p = 0.003; **Figure 4C**). For cohorts aged <70 years, GO favored increased OS (HR 0.92; 95% CI 0.85–1.00, p = 0.044; **Figure 4B**) after sensitivity analyses. CIR of GO was consistently reduced regardless of age stratifications (<60: HR 0.83; 95% CI 0.73–0.93, p = 0.003; ≥60: HR 0.83; 95% CI 0.74–0.93, p = 0.001; <70: HR 0.80; 95% CI 0.73–0.89, p < 0.001; ≥70: HR 0.80; 95% CI 0.64–1.00, p = 0.050; **Figures 4D, E**). For genders, GO consistently favored improved OS and CIR in males (OS: HR 0.85; 95% CI 0.75–0.96, p = 0.010, **Figure 4F**; CIR: HR 0.78; 95% CI 0.66–0.91, p = 0.002, **Figure 4H**), whereas GO showed better EFS in females (HR 0.63; 95% CI 0.49–0.81, p < 0.001; **Figure 4G**) with low heterogeneity.

**Figure 5:**
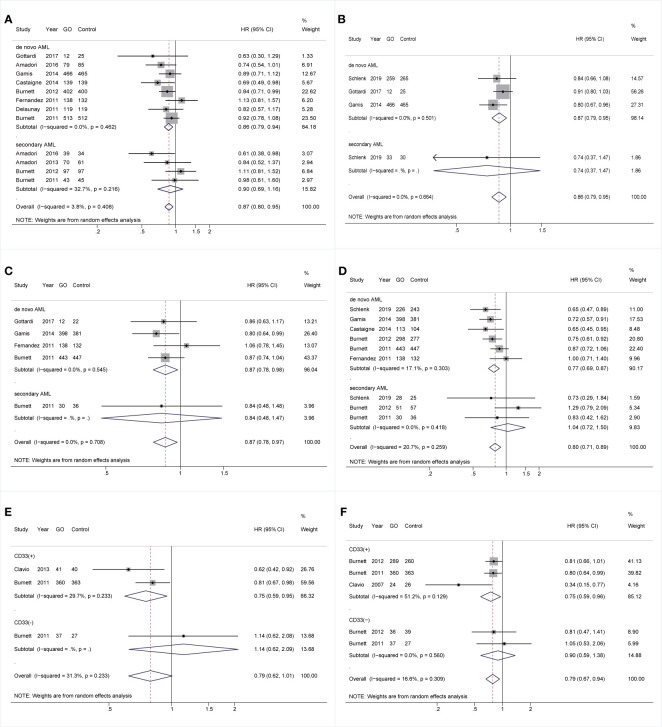
Prognostic subgroup analyses regarding AML types and CD33 expression status. **(A)** OS subgrouped by AML types. **(B)** EFS subgrouped by AML types. **(C)** RFS subgrouped by AML types. **(D)** CIR subgrouped by AML types. **(E)** RFS subgrouped by CD33 expression status. **(F)** CIR subgrouped by CD33 expression status. The diamonds represent the overall summary HR estimates with 95% CI. GO, gemtuzumab ozogamicin; AML, acute myeloid leukemia; OS, overall survival; EFS, event-free survival; RFS, relapse-free survival; CIR, cumulative incidence of relapse; HR, hazard ratio; 95% CI, 95% confidence interval.

Additionally, all survival outcomes were improved in GO in *de novo* AML rather than sAML. In *de novo* AML, GO showed increased OS, EFS, and RFS (OS: HR 0.86; 95% CI 0.79–0.94, p = 0.001; EFS: HR 0.87; 95% CI 0.79–0.95, p = 0.003; RFS: HR 0.87; 95% CI 0.78–0.98, p = 0.017; **Figures 5A–C**) after sensitivity analyses. CIR was consistently reduced in GO (HR 0.77; 95% CI 0.69–0.87, p < 0.001; **Figure 5D**). Finally, in the CD33(+) group, consistently better RFS and CIR were identified (RFS: HR 0.75; 95% CI 0.59–0.95, p = 0.018; CIR: HR 0.75; 95% CI 0.59–0.96, p = 0.021) (**Figures 5E, F**).

### Subgroup Analyses Regarding Treatment Stages, Doses of Gemtuzumab Ozogamicin, and Combined Regimens

**Figure 6:**
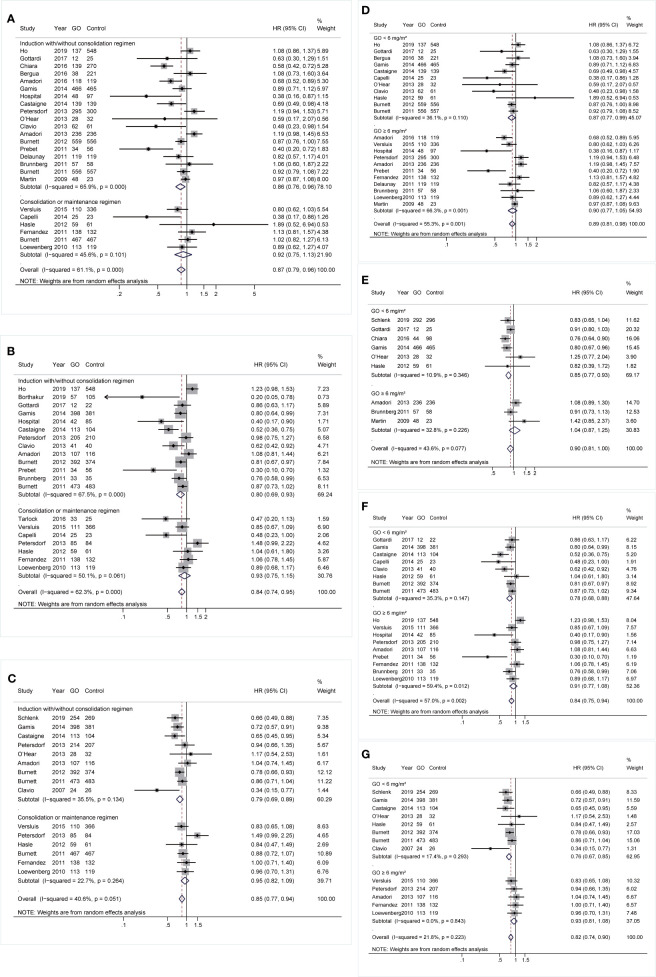
Prognostic subgroup analyses regarding treatment stages and doses of GO. **(A)** OS subgrouped by treatment stages. **(B)** RFS subgrouped by treatment stages. **(C)** CIR subgrouped by treatment stages. **(D)** OS subgrouped by GO doses. **(E)** EFS subgrouped by GO doses. **(F)** RFS subgrouped by GO doses. **(G)** CIR subgrouped by GO doses. The diamonds represent the overall summary HR estimates with 95% CI. GO, gemtuzumab ozogamicin; OS, overall survival; EFS, event-free survival; RFS, relapse-free survival; CIR, cumulative incidence of relapse; HR, hazard ratio; 95% CI, 95% confidence interval.

During induction treatment, GO showed better OS and RFS (OS: HR 0.86; 95% CI 0.76–0.96, p = 0.011; RFS: HR 0.8; 95% CI 0.69–0.93, p = 0.003; **Figures 6A, B**), but the substantial heterogeneity of both was not removed by sensitivity analyses, demonstrating a stable random model. CIR was reduced in GO (HR 0.79; 95% CI 0.69–0.89, p < 0.001; **Figure 6C**) after sensitivity analyses. No prognostic improvement was found in GO when it was only administered in consolidation therapy.

Besides, patients benefited from GO in all survival outcomes at a dose of <6 mg/m^2^ instead of higher doses. When a dose of <6 mg/m^2^ was administered, OS, EFS, and RFS showed pooled HRs of 0.87 (95% CI 0.77–0.99, p = 0.030), 0.85 (95% CI 0.77–0.93, p = 0.001), and 0.78 (95% CI 0.68–0.88, p < 0.001), respectively, after sensitivity analyses (**Figures 6D–F**). GO also consistently favored better CIR (HR 0.76; 95% CI 0.67–0.85, p < 0.001; **Figure 6G**).

**Figure 7:**
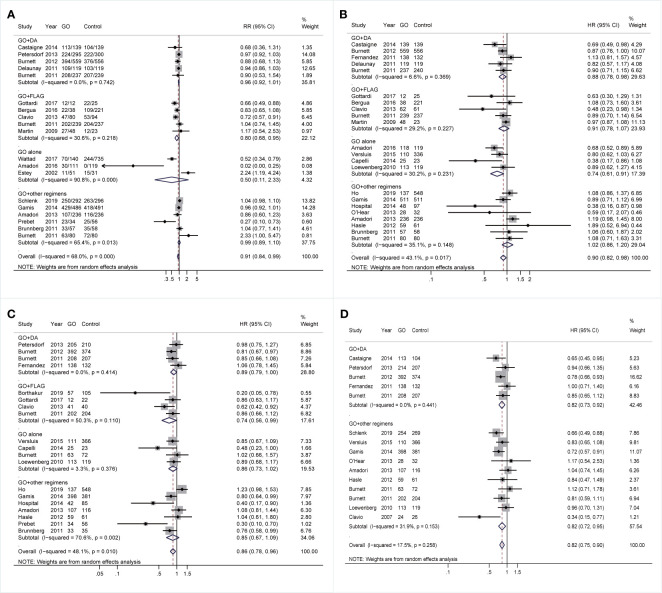
Prognostic subgroup analyses regarding combined regimens of GO. **(A)** CR. **(B)** OS. **(C)** RFS. **(D)** CIR. The diamonds represent the overall summary RR and HR estimates with 95% CI. GO, gemtuzumab ozogamicin; CR, complete remission; OS, overall survival; RFS, relapse-free survival; CIR, cumulative incidence of relapse; RR, relative risk; HR, hazard ratio; 95% CI, 95% confidence interval; DA, daunorubicin plus cytarabine; Ara-C, cytarabine; FLAG, fludarabine, Ara-C, and granulocyte colony-stimulating factor.

Finally, the effectiveness of combined regimens was explored, including GO+DA, GO+FLAG, GO alone, and GO+other regimens. Increased CR of GO+FLAG was found when compared to FLAG (RR 0.80; 95% CI 0.68–0.95, p = 0.009; **Figure 7A**) after sensitivity analyses, followed by improved RFS in GO+FLAG (HR 0.74; 95% CI 0.56–0.99, p = 0.038; **Figure 7C**). Besides, when compared to DA, GO+DA favored increased OS (HR 0.88; 95% CI 0.78–0.98, p = 0.028; **Figure 7B**) and reduced CIR (HR 0.82; 95% CI 0.73–0.92, p = 0.001; **Figure 7D**) after sensitivity analyses. OS was also consistently enhanced in GO monotherapy when compared to the best supportive care (BSC) (HR 0.74; 95% CI 0.61–0.91, p = 0.003; **Figure 7B**). CIR was consistently decreased in GO+other regimens after sensitivity analyses (HR 0.82; 95% CI 0.72–0.95, p = 0.005; **Figure 7D**).

### Network Meta-Analysis for Various Combined Regimens of Gemtuzumab Ozogamicin

**Supplementary Figures 3A–D** displayed the network evidence plots to compare CR, OS, RFS, and CIR between various combined regimens of GO, noting no head-to-head trial in all analyses. Therefore, the summarized data between interventions were produced either from qualified indirect or direct evidence but not from both, and data were unavailable to estimate the inconsistency between direct and indirect comparisons. **Supplementary Figures 3E–H** illustrated the estimated RR and HR for pooled CR, OS, RFS, and CIR, respectively, in NMA. GO monotherapy achieved the best CR (RR, 0.016; 95% Crl, 0.00095–0.25) and OS (HR, 0.71; 95% Crl, 0.51–0.94). GO+DA tended to favor better RFS (HR, 0.84; 95% Crl, 0.63–1.10). GO+ICE+ATRA (all-trans retinoic acid) and GO+DA had a tendency of decreased CIR (GO+ICE+ATRA: HR, 0.66; 95% Crl 0.42–1.00; GO+DA: HR, 0.83; 95% Crl, 0.68–1.00). **Supplementary Figures 3I–L** demonstrated the rank of probability for improved CR, OS, RFS, and CIR, respectively. GO alone favored the highest probability of improved CR and OS, but GO+Ara-C showed the highest probability of improved RFS, and GO+ICE+ATRA (ICE: idarubicin+Ara-C+etoposide) had the highest probability of reduced CIR.

The detailed results of prognostic effect from all treatments are presented in **Supplementary Tables 9–12**. The heterogeneity (**Supplementary Figure 4**) existed among GO+ADE studies in CR (**Supplementary Figure 4A**) and CIR (**Supplementary Figure 4D**), as well as among studies of GO alone and GO+DA in OS (**Supplementary Figure 4B**) and RFS (**Supplementary Figure 4C**).

### Toxicity

In total, early death (defined as induction death or 30-day mortality) and 21 types of toxic effects were analyzed (**Supplementary Table 13**). Among 13 studies, early death did not significantly increase in the GO arm (GO *vs*. non-GO: 8.00% *vs*. 6.57%; RR 1.23; 95% CI 0.91–1.67, p = 0.181), with moderate heterogeneity. However, GO at a dose of ≥6 mg/m^2^ rather than lower doses showed higher early death (GO *vs*. non-GO: 13.3% *vs*. 7.14%; RR 2.01; 95% CI 1.23–3.27, p = 0.005). Besides, GO consistently showed increased risk of hepatic-related adverse effects (GO *vs*. non-GO: 13.70% *vs*. 7.01%; RR 1.29; 95% CI 1.04–1.60, p = 0.02) and a tendency of increased risk for hepatic veno-occlusive disease or sinusoidal obstruction syndrome (VOD/SOS) (GO *vs*. non-GO: 4.19% *vs*. 2.75%; RR 1.56; 95% CI 0.96–2.53, p = 0.072). The dose of ≥6 mg/m^2^ showed a higher tendency of enhanced risk (GO *vs*. non-GO: 4.67% *vs*. 3.00%; RR 2.95; 95% CI 0.56–15.65, p = 0.203) for VOD/SOS when compared to the dose of <6 mg/m^2^ (GO *vs*. non-GO: 3.98% *vs*. 2.50%; RR 1.53; 95% CI 0.85–2.75, p = 0.156). GO group was also associated with a slightly increased risk of bleeding (GO *vs*. non-GO: 22.96% *vs*. 22.49%; RR 1.13; 95% CI 1.02–1.25, p = 0.018). Finally, there was no difference of the risks for the remaining toxic effects between GO and non-GO arms, such as infection (GO *vs*. non-GO: 36.80% *vs*. 37.85%; RR 0.98; 95% CI 0.86–1.11, p = 0.756).

### Sensitivity Analyses and Publication Bias

Sensitivity analyses were conducted if high heterogeneity (p < 0.10) existed. The heterogeneity source has been listed in **Supplementary Tables 4–S8**, related to various combined regimens and various doses of GO, age of patients, cytogenetics, and genetics. In **Supplementary Table 14**, publication bias was found in the analysis of comprehensive RFS (Egger’s test, p = 0.017; Begg’s test, p = 0.049). The funnel plots are shown in **Supplementary Figure 5**.

## Discussion

Due to the limited clinical efficacy of standard chemotherapy for AML, some innovative molecular-targeted therapies, such as GO, have been applied. Up to now, 15 retrospective cohort studies and 15 RCTs comparing therapeutic effects between GO and other regimens have been published. As a result, it is indispensable to integrate all available data for assessing this drug.

This is the biggest systematic review and meta-analysis to evaluate the total treatment evidence regarding GO in AML. GO tended to improve CR, probably resulting in improved survival and declined relapse. Survival benefits of GO were evidently observed in favorable- and intermediate-risk karyotypes. Improved prognosis was found in GO of *NPM1*(+) cohorts and *FLT3-ITD*(-) patients. OS benefits in GO was limited in patients aged ≥70 years, and CIR was reduced regardless of age. Survival benefits were also observed in CD33(+) group instead of CD33(-) patients and in *de novo* AML rather than sAML, but it might be unclear regarding genders. Furthermore, adding GO into induction treatment instead of consolidation alone might produce better survival. Data also showcased more benefits of GO in some survival outcomes at a lower (<6 mg/m^2^) dose of GO instead of ≥6 mg/m^2^. Survival outcomes of various combined regimens with GO were heterogeneous, showing improved OS and CIR in GO+DA, increased OS in GO monotherapy, and longer RFS in GO+FLAG. The NMA also presented inconsistent probability of achieving better survival among different combined regimens. Additionally, GO was related to increased risk of early death at a higher dose (≥6mg/m^2^), hepatic-related adverse effects, and a tendency of higher risk for VOS/SOS. GO was associated with slightly higher risk of bleeding.

Our data did not show significantly improved CR by GO, which was, however, followed by improved survival. These data were consistent with previous meta-analyses (16, 49, 50), but our study comprised more RCTs and considered retrospective cohort studies, leading to more reliable results based on a huge cohort (N = 11,234). The bare benefit of GO on CR might be explained by the lower-dose intensities of chemotherapy in GO compared to control (13, 28, 29, 38, 46). However, our data displayed increased CR of GO in *FLT3-ITD*(-) subgroup, all studies (8, 9, 39) of which utilized the same combined regimen of GO as control. After excluding the factor of different intensity of chemotherapy between GO and non-GO arms, *FLT3-ITD* mutation might unfavorably affect response to GO.

Besides, the results of survival outcomes were heterogeneous, which might be settled by various subgroups. Cytogenetic risks might play a role in affecting GO benefit, especially in favorable and intermediate-risk karyotypes. This finding, consistent with preceding meta-analyses (16, 49, 50), indicated that GO benefit might be limited to favorable- and/or intermediate-risk cytogenetic groups but requires to be further estimated in RCTs. Another adverse factor frequently affecting prognosis in AML was *FLT3-ITD* mutation (8), which also affected the therapeutic effectiveness of GO, showing that the benefit of GO was observed only in *FLT3-ITD*(-) patients, resulting from better CR. As for *NPM1* mutation, a mutation favoring better survival (14), increased RFS was found in the GO arm of the *NPM1*(+) cohort, but OS and CIR were improved in the GO arm regardless of the *NPM1* mutational status, totally showing benefits of GO in the *NPM1*(+) group.

Additionally, since GO was a CD33-targeting antibody (6), GO also contributed to more survival benefits in CD33(+) AML in our study. Furthermore, enhanced OS and RFS of GO were restricted to cohorts aged ≥60 years, but better OS was observed in subjects aged <70 years in a larger cohort, and CIR was not affected by the threshold of 60 years old, indicating a total better survival achieved in patients aged <70 years. Besides, survival benefits of GO were found in *de novo* AML instead of sAML, another high-risk factor resistant to treatment (38).

The plausible explanation underlying these data was that GO administration might be more beneficial in chemosensitive (favorable- and intermediate-risk cytogenetics, younger age, *NPM1* mutation, *de novo* AML) patients and not beneficial in chemoresistant (inferior cytogenetics, the elderly cohort, *FLT3-ITD* mutation, sAML) cohorts. Further investigations of GO in younger patients, *FLT3-ITD*(-) cohorts, and those with low-/intermediate-risk karyotypes and *de novo* AML warrant future estimation. A special RCT focusing on *NPM1* mutation was initially finished (NCT01237808) (14), showing improved survival (EFS: HR 0.83; 95% CI 0.65–1.04; CIR: HR 0.66; 95% CI 0.49–0.88).

Furthermore, our study displayed the greatest amount of evidence of survival benefits resulting from GO administration in induction regimens rather than only in the consolidation stage. A possible explanation underlying this result seems like an effective adjunct in the induction treatment of AML, and the early GO treatment may prevent relapse and prolong survival. As a consequence, suggested optimization of induction trials warrants the highest attention. Additionally, this study showed that a GO dose of <6 mg/m^2^ favored better survival and lower relapse but no survival advantage at a dose of ≥6 mg/m^2^. Consistently, five RCTs (8–10, 14, 35) prescribing a GO dose of <6 mg/m^2^ did not show a difference in early death between GO and controls, whereas some RCTs (13, 38) with a dose of ≥6 mg/m^2^ reported higher early death rates with GO. This proposed that lower doses, perhaps <6 mg/m^2^, in this setting might be safer and inevitably related to lower toxicity.

Besides, we did not only analyze different therapeutic effects of combined regimens with GO in meta-analysis but also in NMA. In total, GO alone, GO+FLAG, and GO+DA subgroups supported better prognosis in meta-analysis, whereas the NMA indicated GO alone favored the highest probability of improved CR and OS, but GO+Ara-C harbored the highest probability of improved RFS, and GO+ICE+ATRA had the highest probability of decreased CIR. In addition to the survival benefit of GO alone regarding BSC as control, other survival benefits came from various combined chemotherapies, probably indicating the identified advantages of adding GO into chemotherapy but not yet identifying which combination was the best, which should be further explored in RCTs.

Finally, it was not surprising that our meta-analysis showed increased risk of early death at a higher dose (≥6 mg/m^2^), hepatic-related adverse effects, and VOS/SOS in the GO group, as previously shown in other meta-analyses (16, 49, 50). Besides, the GO group was associated with a slightly higher risk of bleeding, which can be timely discovered and treated in the clinic.

There were several advantages of this meta-analysis. Firstly, we performed the biggest meta-analysis to provide the most up-to-date evidence of GO in AML, including all RCTs and retrospective cohort research with available data. Secondly, the inclusive high-quality research ensured the reliability of this meta-analysis. Thirdly, we did a comprehensive subgroup analysis, such as mutations, *de novo* AML/sAML, and combined regimens that were not reported in published meta-analyses, and identified several subsets of patients who would mostly benefit from this drug, which, of course, require to be further estimated in RCTs. Fourthly, a comprehensive NMA was conducted to explore the best combined regimen with GO, which was not done in other meta-analyses. However, like most meta-analyses, our analysis was based on published summary estimates rather than individual patient data. Consequently, the merged survival curves could not be produced to explore patient-level factors, particularly in several particularly targeted subgroups for GO identified in this study [e.g., patients aged <70 years, cases with low- and intermediate-risk karyotypes, *FLT3-ITD*(-) cohorts, *NPM1*(+) patients, *de novo* AML with positive expression of CD33, and patients receiving GO combined with DA or FLAG].

In conclusion, our study showed that GO could improve prognosis in AML patients, especially for those aged <70 years, with *de novo* AML, with positive expression of CD33, with *NPM1* mutation, without *FLT3-ITD* mutation, and with low-/intermediate-risk karyotypes. A lower dose of GO (<6 mg/m^2^) and using GO in induction stage rather than only in consolidation therapy might lead to less early death, better survival, and lower relapse. Combining GO with other chemotherapies probably favored better prognosis when compared to chemotherapy alone. Further studies involved in such subgroups above are warranted, and more head-to-head RCTs are needed to directly identify the best combining regimen with GO.

## Data Availability Statement

The raw data supporting the conclusions of this article will be made available by the authors, without undue reservation.

## Author Contributions

QX and LY contributed to conception and design of the study. QX and SH collected data from the databases. QX and SH performed the statistical analyses. QX and LY wrote the first draft of the manuscript. All authors contributed to the article and approved the submitted version.

## Funding

This work was supported by the Chinese National Major Project for New Drug Innovation (2019ZX09201002003), National Natural Science Foundation of China (82030076, 82070161, 81970151, 81670162, and 81870134), Shenzhen Science and Technology Foundation (JCYJ20190808163601776, JCYJ20200109113810154), and Shenzhen Key Laboratory Foundation (ZDSYS20200811143757022).

## Conflict of Interest

The authors declare that the research was conducted in the absence of any commercial or financial relationships that could be construed as a potential conflict of interest.

## Publisher’s Note

All claims expressed in this article are solely those of the authors and do not necessarily represent those of their affiliated organizations, or those of the publisher, the editors and the reviewers. Any product that may be evaluated in this article, or claim that may be made by its manufacturer, is not guaranteed or endorsed by the publisher.
