# Supplementary material for: Clinical Benefits and Safety of Gemtuzumab Ozogamicin in Treating Acute Myeloid Leukemia in Various Subgroups: An Updated Systematic Review, Meta-Analysis, and Network Meta-Analysis
Source: Front Immunol. 2021 Aug 16;12:683595. doi: 10.3389/fimmu.2021.683595 (PMC8415423; doi:10.3389/fimmu.2021.683595)
Supplement: Supplementary file 1 [file DataSheet_1.docx]

Supplementary Material

# Supplementary Tables

**Supplementary Table 1: The preferred reporting items for systematic reviews and meta-analyses (PRISMA)**

| Section/topic | # | Checklist item | Reported on page # |
| --- | --- | --- | --- |
| **TITLE** | | | |
| Title | 1 | Identify the report as a systematic review, meta-analysis, or both. | 1 |
| **ABSTRACT** | | | |
| Structured summary | 2 | Provide a structured summary including, as applicable: background; objectives; data sources; study eligibility criteria, participants, and interventions; study appraisal and synthesis methods; results; limitations; conclusions and implications of key findings; systematic review registration number. | 1, 2 |
| **INTRODUCTION** | | | |
| Rationale | 3 | Describe the rationale for the review in the context of what is already known. | 2 |
| Objectives | 4 | Provide an explicit statement of questions being addressed with reference to participants, interventions, comparisons, outcomes, and study design (PICOS). | 2, 3 |
| **METHODS** | | | |
| Protocol and registration | 5 | Indicate if a review protocol exists, if and where it can be accessed (e.g., Web address), and, if available, provide registration information including registration number. | PROSPERO (CRD42020158540) |
| Eligibility criteria | 6 | Specify study characteristics (e.g., PICOS, length of follow-up) and report characteristics (e.g., years considered, language, publication status) used as criteria for eligibility, giving rationale. | 3 |
| Information sources | 7 | Describe all information sources (e.g., databases with dates of coverage, contact with study authors to identify additional studies) in the search and date last searched. | 3 |
| Search | 8 | Present full electronic search strategy for at least one database, including any limits used, such that it could be repeated. | 3 |
| Study selection | 9 | State the process for selecting studies (i.e., screening, eligibility, included in systematic review, and, if applicable, included in the meta-analysis). | 3 |
| Data collection process | 10 | Describe method of data extraction from reports (e.g., piloted forms, independently, in duplicate) and any processes for obtaining and confirming data from investigators. | 3 |
| Data items | 11 | List and define all variables for which data were sought (e.g., PICOS, funding sources) and any assumptions and simplifications made. | 3 |
| Risk of bias in individual studies | 12 | Describe methods used for assessing risk of bias of individual studies (including specification of whether this was done at the study or outcome level), and how this information is to be used in any data synthesis. | 3 |
| Summary measures | 13 | State the principal summary measures (e.g., risk ratio, difference in means). | 3 |
| Synthesis of results | 14 | Describe the methods of handling data and combining results of studies, if done, including measures of consistency (e.g., I^2^) for each meta-analysis. | 4 |
| Risk of bias across studies | 15 | Specify any assessment of risk of bias that may affect the cumulative evidence (e.g., publication bias, selective reporting within studies). | 3 |
| Additional analyses | 16 | Describe methods of additional analyses (e.g., sensitivity or subgroup analyses, meta-regression), if done, indicating which were pre-specified. | 4 |
| RESULTS | | | |
| Study selection | 17 | Give numbers of studies screened, assessed for eligibility, and included in the review, with reasons for exclusions at each stage, ideally with a flow diagram. | 4 |
| Study characteristics | 18 | For each study, present characteristics for which data were extracted (e.g., study size, PICOS, follow-up period) and provide the citations. | 4, 5 |
| Risk of bias within studies | 19 | Present data on risk of bias of each study and, if available, any outcome-level assessment (see Item 12). | 5 |
| Results of individual studies | 20 | For all outcomes considered (benefits or harms), present, for each study: (a) simple summary data for each intervention group and (b) effect estimates and confidence intervals, ideally with a forest plot. | 5, 6, 7 |
| Synthesis of results | 21 | Present results of each meta-analysis done, including confidence intervals and measures of consistency. | 5, 6, 7 |
| Risk of bias across studies | 22 | Present results of any assessment of risk of bias across studies (see Item 15). | 8 |
| Additional analysis | 23 | Give results of additional analyses, if done (e.g., sensitivity or subgroup analyses, meta-regression [see Item 16]). | 5, 6, 7 |
| DISCUSSION | | | |
| Summary of evidence | 24 | Summarize the main findings including the strength of evidence for each main outcome; consider their relevance to key groups (e.g., health care providers, users, and policy makers). | 8, 9, 10 |
| Limitations | 25 | Discuss limitations at study and outcome level (e.g., risk of bias), and at review level (e.g., incomplete retrieval of identified research, reporting bias). | 10 |
| Conclusions | 26 | Provide a general interpretation of the results in the context of other evidence, and implications for future research. | 10 |
| FUNDING | | | |
| Funding | 27 | Describe sources of funding for the systematic review and other support (e.g., supply of data); role of funders for the systematic review. | 10 |

**Supplementary Table 2: Study characteristics in 15 randomized controlled trials and 15 retrospective cohort studies**

| **Author** | **Published year** | **Journal** | **Region** | **Study type** | **Trial** | **Entry criteria** | **Period** | **Median age (ranges) or Mean age (±SE)** | **Cohort size** | **GO treatment stage** | **Dose of GO** | **Concomitant therapy** | **N of GO** | **Control group** | **N of control** |
| --- | --- | --- | --- | --- | --- | --- | --- | --- | --- | --- | --- | --- | --- | --- | --- |
| Richard F. Schlenk | 2019 | Journal of Clinical Oncology | Austria, Germany | A prospective, randomized, open-label, multicenter, phase III trial (AMLSG 09-09) | NCT00893399 | NPM1 (+) AML patients aged ≥ 18 years | May 2010 - September 2017 | Total: 58.7 (18.4-82.3) years; GO: 58.6 (18.4-82.3) years; Control: 58.7 (20.9-80.2) years | 588 | Induction and consolation regimen | Induction: 3 mg/m^2^ IV on day1; Consolidation: GO (3 mg/m^2^ IV on day1) in first consolidation therapy. | Idarubicin (12 mg/m^2^ IV on day1, 3, 5 [in cycle 2 and for patients > 60 years, reduced to day1-3]), Ara-C (100 mg/m^2^ IV on day1-7), and etoposide (100 mg/m^2^ IV on day1-3 [in cycle 2 and for patients > 60 years, reduced to day1-3]; ICE) plus ATRA (45 mg/m^2^ orally [PO] on day6-8 and 15 mg/m^2^ PO on day9-21). Etoposide was reduced from 3 to 2 days in cycle 2. Patients received 3 cycles of HiDAC + ATRA, with Ara-C (18-60 years, 3 g/m^2^/12 hours, day1-3; > 60 years, 1 g/m^2^/12 hours, day1-3), ATRA (15 mg/m^2^/d PO, day4-21), and pegfilgrastim (6 mg subcutaneously) on day8. | 292 | As shown in the concomitant therapy | 296 |
| Vincent T. Ho | 2019 | Biol Blood Marrow Transplant | USA | A retrospective, matched-cohort analysis | - | Adult AML patients with allo-HSCT | 2008 - 2011 | GO: 42 (18-73) years; Control: 38 (18-74) years | 685 | Induction and consolation regimen | Median (range) total GO dose: 9.5 (3.0-33.0) mg | Not clear | 137 | Not clear | 548 |
| Gautam M. Borthakur | 2019 | Blood, Am Soc Hematology | USA | A retrospective study | - | Patients with CBF-AML | April 2007 - January 2018 | Total: 49 (19-78) years, 15% older than 65 years | 162 | Induction regimen | GO: at 3 mg/m^2^ on day 1 in induction and on day 2 of the planned 6 post-remission cycles | FA: fludarabine 30 mg/m^2^ daily × 5days and Ara-C 2 gm/ m^2^ × 5 days starting 3.5 h after completion of that day’s fludarabine. In the FLAG, patients received G-CSF μgm/kg subcutaneously daily on day1-5, in addition to FA. | 57 | FLAG-IDA, in combination with idarubicin at 6 mg/m^2^ on day3-4 in induction regimen. | 105 |
| M Wattad | 2017 | Leukemia | Europe, Australia | A retrospective study | NCT00146120 and NCT00151255 | 845 patients had refractory AML after first induction therapy and n= 44 as well as n = 136 patients initially achieving a CR/CRi or PR after first induction therapy, respectively, relapsed after second induction therapy and were therefore also categorized as induction failure. | 1993 - 2009 | Total: 55 (16–84) years; GO: 48.5 (22.2–71.7) years; | 875 | Reinduction therapy in the AMLSG 07-04 study | Not clear | High-dose Ara-C (0.5 g/m^2^ twice a day, days 1–3) and etoposide followed by ATRA (A-HAE) | 140 | Induction: ICE (idarubicin 12 mg/m^2^ IV day1-3, Ara-C 100 mg/m^2^ IV day1–5, etoposide 100 mg/m^2^ IV day1-3); IA (idarubicin, 12 mg/m^2^ IV day1–3; Ara-C, 100 mg/m^2^ IV day1–5). Salvage regimen: HiDAC (3 g/m^2^, BID, day1–3) and mitoxantrone (HAM), for age >55 years; Sequential-HAM (S-HAM, Ara-C 2 g/m^2^ BID, day1, 2, 8 and 9) for age 18–55 years; HAM followed by ATRA (A-HAM); HiDAC (0.5 g/m2 BID, day1–3) and etoposide followed by ATRA; FLAG and FLAG-IDA; ‘7+3’- HiDAC- based therapy; Phase-I/II trials: Fludarabine + A-HAM; Others. 87 patients received allo-HSCT without prior chemotherapy. | 735 |
| Michele Gottardi | 2017 | Hematology Reports | Italy | A retrospective study | MyFLAI | Patients with CBF-AML | 2003 - 2010 | GO: 46.3 (29-67) years; Control: 41.3 (18-66) years | 37 | Induction regimen | Induction: GO 3 mg/m^2^ on day 6 | Induction: FLAI, Fludarabine 30mg/m^2^ on day1-5; Ara-C 2 g/m^2^ on day1-5; Idarubicin 10 mg/m^2^ on days 1,3,5, consolidation: consolidated with two HiDAC based cycles (overall dose 24 g/m^2^/cycle). | 12 | As shown in the concomitant therapy | 25 |
| Katherine Tarlock | 2016 | Clinical Cancer Research | USA | A retrospective study, including the phase III study AAML03P1 and phase III trial AAML0531 | NCT00070174 and NCT00372593 | Pediatric patients aged 1 month to 30 years with de novo FLT3-ITD (+) AML | December 2003 - November 2005, and August 2006 - June 2010 | GO: 12.1 (1.6–20.9) years; Control: 13.2 (0.7–20.4) years | 183 | Induction regimen | GO (dose 3 mg/m^2^) administered once during induction course I on day 6 and again during intensification course II on day 7 | Induction I: HiDAC IV BID on day1-10; daunorubicin IV on day1, 3, 5; etoposide IV on day1-5; II: Ara-C IV BID on day1-8; Ara-C on day 1; and daunorubicin and etoposide as in induction I; Intensification course I: Ara-C IV BID on day1-5; Ara-C as in induction II; and etoposide IV on day1-5; II: Ara-C IV BID on day1-4; Ara-C as in induction II; mitoxantrone IV on day3-6; | 112 | As shown in the concomitant therapy | 71 |
| Sartor, Chiara | 2016 | Haematologica | Italy | A retrospective study | - | Patients with newly diagnosed AML | 1997 - 2014 | GO: 53 (19-74) years; Control: 52 (17-52) years | 409 | Induction regimen | MyFLAI or MyAIE schedules | MyFLAI or MyAIE schedules | 139 | FLAI, FLAN, FLAG, 3+7 or DAE | 270 |
| Juan M. Bergua | 2016 | British Journal of Haematology | Spain | A retrospective study (the PETHEMA Spanish AML) | NCT02006004 | Patients with de novo or secondary non‐M3 AML | 1999 - 2012 | Total: 54 (16–76) years | 259 | Induction regimen | GO dose was 3 mg/m^2^ on day 1 | FLAG‐IDA: G‐CSF priming at dose of 300 μg on day1–3; idarubicin 10 mg/m^2^ on day2–4, fludarabine 30 mg/m^2^ on day2–5 and Ara-C 2g/m^2^ on day2–5 (4 h after fludarabine infusion) | 38 | As shown in the concomitant therapy | 221 |
| Sergio Amadori | 2016 | Journal of Clinical Oncology | Europe | An open-label, phase III trial, the Randomized EORTC-GIMEMA AML-19 Trial | - | AML ≥ 75 years, and 61-75 years with a WHO performance score > 2 not receiving standard chemotherapy | November 2004 - May 2013 | Total: 77 (62-88) years; GO: 77 (62-88) years; BSC: 77 (66-88) years | 237 | Induction and consolation regimen | A single induction course: two IV infusions, administered at 6 mg/m^2^ on day 1 and 3 mg/m^2^ on day 8. | Null. | 118 | BSC included blood product transfusions, antimicrobials, and other symptomatic therapies. | 119 |
| Jurjen Versluis | 2015 | The Lancet Haematology | Europe | A retrospective study, including HOVON-SAKK trials | AML42/42A, AML43, AML81, and AML92 | Patients ≥ 60 years with newly diagnosed AML | May 3th 2001 - February 5th 2010 | - | 476 | Consolidation and maintenance regimen | GO: 6 mg/m² every 4 weeks for three cycles | Not clear | 110 | Not clear | 366 |
| Alan S. Gamis | 2014 | Journal of Clinical Oncology | USA, Australia, Canada, New Zealand, Puerto Rico, Switzerland | A randomized open -label phase 3 clinical trial | Children’s Oncology Group Trial AAML0531, NCT00372593 | Pediatric patients aged 1 month to 29.99 years, who had previously untreated primary AML | August 2006 - June 2010 | Total: 9.7 (0.003-29.8) years; GO: 9.9 (0.02-29.4) years; Control: 9.5 (.003-29.8) years | 1022 | Induction and consolation regimen | GO, 3 mg/m^2^, once on day 6 of induction course 1 and once on day 7 of intensification course 2 | Induction course 1 (Ara-C 100 mg/m^2^ BID, day1-10; Daunomycin, 50 mg/m^2^ IV, day1,3,5; Etoposide 100 mg/m^2^ IV day1-5) and intensification course 2 (Mitoxantrone 12 mg/m^2^ IV, day3-6; Ara-C 1g/m^2^ BID IV day1-4) | 511 | As shown in the concomitant therapy | 511 |
| Marie-Anne Hospital | 2014 | Blood | Europe | A retrospective study | - | 1) CBF-AML was diagnosed in first relapse; 2) previously treatment with ≥ one course of anthracycline, plus Ara-C-based induction therapy; 3) no treatment with GO or HSCT | 1994 - 2011 | Total: 43 (16-76) years | 145 | Reinduction regimen | GO was 6 mg/m^2^ in 31 cases and fractionated doses (3 mg/m^2^ on days 1, 4, 7) in 4 cases. In 13 cases with Ara-C + anthracycline; GO was 9mg/m^2^ in 7 cases, fractionated 3mg/m^2^ days 1, 4, 7 in 1 patient, and 6 mg/m^2^ in 5 cases. | HiDAC alone without anthracycline;  Standard dose of Ara-C with additional anthracycline. | 48 | HiDAC alone without anthracycline (N = 53);  Standard dose of Ara-C with additional anthracycline (N = 44). | 97 |
| Sylvie Castaigne | 2014 | Lancet Oncology | France | A randomized, open-label, multicenter, phase III clinical trial, ALFA-0701 study | EuduraCT n.: 2007-002933-36; NCT00927498 | Previously untreated AML patients, aged 50–70 years | January 2008 - November 2010 | Total: 62·2 (58·5–66·3) years; GO: 62·8 (59·3–66·8) years; Control: 61·7 (57·4–65·6) years | 278 | Induction and consolation regimen | Induction: GO (3 mg/m² [maximum of 5 mg] IV infused on days 1, 4, and 7; Consolidation: GO IV (3 mg/m² on day 1) | Induction: 3+7 induction course of IV daunorubicin (60 mg/m² on day1-3) and IV Ara-C (200 mg/m² as continuous infusion for 7 days); Consolidation: two courses of IV daunorubicin (60 mg/m² for 1 day [first course] or 2 days [second course]) plus IV Ara-C (1 g/m² per 12 h, infused over 2 h on day1–4). | 139 | As shown in the concomitant therapy | 139 |
| Debora Capelli | 2014 | Biol Blood Marrow Transplant | Italy | A randomized, phase II prospective study | R000014052 | Elderly AML patients in CR1 after induction therapy | - | GO: 68 (61-76) years; Control: 67 (60-77) years | 48 | Post-consolidation regimen in CR | GO: 3mg/m^2^ IV, every 28 days for 3 months, followed by 3 infusions at 3-month intervals. | Null. | 25 | Autologous Transplantation | 23 |
| Stephen H. Petersdorf | 2013 | Blood | USA, Canada, Sweden | A randomized open-label phase 3 trial, SWOG S0106 | NCT00085709 | Patients with AML, aged 18 to 60 years | August 2004 - August 2009 | DA+GO: 47 (18-60) years; DA: 48 (18-60) years | 595 | Induction regimen | GO 6 mg/m^2^ by 2-hour IV infusion on day 4 | DA: daunorubicin 45 mg/m2 by IV push on day1-3, Ara-C 100 mg/m^2^ by continuous IV infusions on day1-7 | 295 | Daunorubicin 60 mg/m^2^ by IV push on day1-3 and Ara-C 100 mg/m^2^ by continuous IV infusion on day1-7 | 300 |
| Carol O’Hear | 2013 | Cancer | USA | A randomized, open-label, phase III trial | AML02 multicenter trial, | Patients aged 2 days-21.4 years, with non-M3 AML, without Down syndrome | 2002 - 2008 | 9.1 years (2 days-21.4 years) | 60 | Induction regimen | Induction 2: GO 3 mg/m^2^; Patients with an MRD 0.1% after induction 2 were given GO at a dose of 6 mg/m^2^ as induction 3. | Patients randomly received daunorubicin (50 mg/m^2^ on days 2, 4, 6) and etoposide (100 mg/m^2^ on day2–6) plus either HiDAC (3 g/m^2^ every 12 hours on days 1, 3, 5) or low-dose Ara-C (100 mg/m^2^ every 12 hours. During induction 2, patients received low-dose Ara-C, daunorubicin, and etoposide. | 28 | As shown in the concomitant therapy | 32 |
| Marino Clavio | 2013 | Ann Hematol | Italy | A prospective clinical trial + historical control | FLAI-GO trial | Untreated CD33+ (defined by flow cytometric method) non-M3 AML patients aged 60 or over | May 2004 - September 2009 | - | 189 | Induction and consolation regimen | Induction: GO (3 mg/m^2^) was administered at day 4. Consolidation: GO (3 mg/m^2^) | Induction: FLAI: fludarabine (30 mg/m^2^, 1h, day1–3), followed 4 h later by Ara-C (1 g/m^2^, 4h, day1–3) and idarubicin (5 mg/m^2^, 30min, day1–3). Consolidation: Ara-C, 1 g/m^2^ every 12 h for three times, every 3 months. | 85 | As shown in the concomitant therapy | 104 |
| Sergio Amadori | 2013 | Journal of Clinical Oncology | Europe | A randomized, open-label, phase III trial by the EORTC and GIMEMA Consortium | AML-17 | Patients aged 61 to 75 years with newly diagnosed AML | September 2002 - August 2007 | Total: 67 (60-75) years; GO: 67 (61-75) years, Control: 68 (61-75) years | 472 | Induction and consolation regimen | Induction: 6mg/m^2^ IV on day1 and 15 before the concomitant chemotherapy; Consolidation: 3mg/m^2^ IV on day 0. | Induction: MICE (mitoxantrone 7mg/m^2^ IV on day1,3,and 5; etoposide 100 mg/m^2^ IV on day1-3; and Ara-C 100mg/m^2^/day IV infusion on day1-7); Consolidation: ICE (idarubicin 8 mg/m^2^ IV on days 1, 3 and 5; Ara-C 100mg/m^2^/day IV in fusion on day1-5; and etoposide 100mg/m^2^ IV on day1-3) | 236 | As shown in the concomitant therapy | 236 |
| Henrik Hasle | 2012 | Blood | Denmark, Finland, Iceland, Norway, Hongkong and Sweden | A randomized open-label phase 3 study | NCT00476541, NOPHO-AML 2004 study for children with AML | High-risk children AML patients, defined as having poor response to induction (> 15% blasts after AIET or no remission after second induction) or the presence of MLL rearrangements other than t(9;11)(p21;q23) | 2004 - 2010 | - | 120 | Consolidation regimen | GO was administered as a 2-hour infusion of 5 mg/m^2^ at least 4 weeks after the last consolidation course and repeated after an interval of 3 weeks. | High-risk patients were eligible for HSCT after completing 3 courses but before the last consolidation. Induction regimen: AIET, Ara-C 200 mg/m^2^ infusion day1-4, 6-thioguanine 100 mg/m^2^ BID day1-4, etoposide 100 mg/m^2^ day1-4, idarubicin 12 mg/m^2^ days 2, 4, and 6. AM, Ara-C 100 mg/m^2^ infusion day1-5, mitoxantrone 10 mg/m^2^ day1-3. Consolidation: HA1M, Ara-C 1 g/m^2^ BID day1-3, mitoxantrone 10 mg/m^2^ day3-5. HA2E, Ara-C 2 g/m^2^ BID day1-3, etoposide 100 mg/m^2^ day2-5. HA3, Ara-C 3 g/m^2^ BID day1-3. | 59 | As shown in the concomitant therapy | 61 |
| Alan K. Burnett | 2012 | Journal of Clinical Oncology | United Kingdom and Denmark | A phase III randomized multicenter study | AML16 | Patients with AML or high-risk MDS with > 10% blasts, aged ≥ 60 years; And patients < 60 years and not suitable for the trial including HiDAC. | December 4th 2006 - July 2nd 2010 | Total: 67 (51-84) years; GO: 67 (51-84) years; Control: 67 (57-80) years | 1115 | Induction regimen | GO 3mg/m^2^ on day1 | Daunorubicin + Ara-C 3 + 10: daunorubicin 50 mg/m^2^/day on day1, 3, and 5 plus Ara-C 100 mg/m^2^ on day1-10, every 12 hours; daunorubicin + clofarabine: daunorubicin 50mg/m^2^/day on days 1, 3, and 5, plus clofarabine 20mg/m^2^/day on day1-5. | 559 | As shown in the concomitant therapy | 556 |
| Thomas Prebet | 2011 | Cancer | France, the Institut Paoli‐Calmettes | A retrospective study | - | AML patients relapsed after induction regimen with CR1 duration ≥ 3 months, treated with intermediate- to high-dose Ara-C | 2000 - 2010 | Total: 50 (24-71) years | 90 | Induction and consolation regimen | The average dose of GO was 6 mg/m^2^/d (range, 3-9 mg/m^2^/d) | Ara-C >500 mg/m^2^ alone; MEC: Ara-C 1000 mg/m^2^/d, day1-5; Mitoxantrone 8 mg/m^2^/d, day1-5; Etoposide 100 mg/m^2^/d, day1-5; Ara-C 2g/m^2^/d, day1-5, Etoposide 100 mg/m^2^/d, day1-5; And Ara-C 2g/m^2^/d, day1-5, Topotecan 1.5 mg/m^2^/d, day1-5; Others. | 34 | As shown in the concomitant therapy | 56 |
| Hugo F. Fernandez | 2011 | Blood | USA, Israel | A randomized open-label phase 3 trial (ECOG trial E1900) | NCT00049517 | De novo untreated AML aged from 17 to 60 years | December 2002 - November 2008 | GO: 48 (18-60) years; Control: 47 (18-60) years | 270 | Consolidation regimen | 6 mg/m^2^, IV | Patients were randomized to receive Ara-C 100 mg/m^2^/d IV for 7 days plus IV daunorubicin daily for 3 days at 45 or 90 mg/m^2^. The second cycle was based on the daunorubicin (45 mg/m^2^) for 3 days. Consolidation: All patients received 2 cycles of HiDAC (3 g/m^2^ IV over 3 h every 12 hours every other day for a total of 6 doses), followed by sargramostim 250 ug/m^2^ until recovery of blood counts. | 138 | As shown in the concomitant therapy | 132 |
| Jacques Delaunay | 2011 | Blood | Europe | A phase III prospective randomized trial | Results of the GOELAMS AML 2006 IR Study | De novo AML patients aged 18-60 years, with intermediate karyotype | 2007 - 2010 | Total: 50 (18–60) years | 238 | Induction and consolation regimen | GO 6mg/m^2^ | 3+7 regimen and a first MidAc intensive consolidation course (mitoxantrone and intermediate doses of Ara-C) | 119 | As shown in the concomitant therapy | 119 |
| U. Brunnberg | 2011 | Annals of Oncology | Germany | A randomized phase II trial |  | De novo or secondary AML aged 60 years and more, and CD33+ | June 2005 - June 2009 | GO: 69 (60–83) years; Control: 68 (60–78) years | 115 | Induction regimen | GO (6 mg/m^2^ IV, day 1 and 4 mg/m^2^ IV, day 8) | Ara-C (100 mg/m^2^/24 h IV as continuous infusion, day1–7) | 57 | Ara-C (100 mg/m^2^/24 h IIV, day1–7) and daunorubicin (60 mg/m^2^ IV day3–5) | 58 |
| Alan K. Burnett | 2011 | Journal of Clinical Oncology | the United Kingdom, Denmark, and New Zealand | A phase III randomized multicenter study | the MRC AML15 Trial | De novo or secondary non-M3 untreated AML, aged 15 years and more, with a negative pregnancy test and normal liver function | July 2002 - March 2009 | Induction regimen: GO: 49 (0-69) years; Control: 49(2-71) years; Consolidation regimen: GO: 46 (0-69) years; Control: 46 (0-68) years | Induction: 1113; Consolidation: 948 | Induction regimen or consolidation regimen | Induction regimen: GO 3mg/m^2^ on day1; Consolidation regimen: GO 3mg/m^2^ on day1 | Induction: DA: daunorubicin 50mg/m^2^ d1, 3, 5 + Ara-C 100mg/m^2^, d1-10 every 12h; FLAG-IDA: fluduarabine 30mg/m^2^ IV d2-6 + Ara-C 2g/m^2^ over 4h after fluduarabine on d2-6 + G-CSF 263µg daily d1-7; ADE: daunorubicin 50mg/m^2^ d1,3,5; Ara-C 100mg/m^2^ d1-10 every 12h; etoposide 100mg/m^2^ d1-5; Consolidation: Ara-C 1.5 or 3g/m^2^ d IV over 4h hourly on d1,3,5; MACE: amsacrine 100mg/m^2^ d1-5, Ara-C 200mg/m^2^ d1-5; etoposide 100mg/m^2^ d1-5. | Induction: 556; Consolidation: 473 | As shown in the concomitant therapy | Induction: 557; Consolidation: 475 |
| Antonella Poloni | 2010 | British Journal of Haematology | Italy | A retrospective study | - | Non-M3 AML aged 60 years and over | June 1999 - December 2007 | Total: 70 (61–76) years | 38 | Consolidation regimen | 3 mg/m^2^ three times monthly | Null. | 13 | Autologous stem cell transplantation (ASCT); Chemotherapy (CHT). | ASCT:19  CHT: 6 |
| Bob Loewenberg | 2010 | Blood | Europe | A randomized multicenter phase 3 study | ISRCTN77039377 | Non-M3 AML ≥ 60 years, in CR after 2 cycles of induction therapy | October 27th 2000 - June 9th 2006 | GO: 67 (60-77) years; Control: 66 (60-78) years | 232 | Post-consolidation regimen in CR | 3 cycles of GO at 6 mg/m^2^ per 2-hour infusion at 4-week intervals | Null. | 113 | Null. | 119 |
| Mark R. Litzow | 2010 | British Journal of Haematology | USA | A phase II randomized trial | NCT00005962 | Non-M3 adult refractory AML patients or relapsed patients within 12 months after CR1 | July 2000 - May 2002 | GO: 60 (27-75) years; DA: 52 (27-85) years; Others: 53 (25-78) years | 82 | Induction regimen | GO 6 mg/m2 IV, day 5 | Ara-C 1 gm/m^2^/ day IV, day1–4 | 26 | Arm 1: Ara-C 1 g/m^2^/ day IV, day1–4, daunorubicin 135 mg/m^2^/day IV, day1–3; Arm 2: Cyclophosphamide 300 mg/m^2^ every 12 h IV, day1–3, Ara-C 1 g/m^2^/ day IV, day2–6, Topotecan 1.5 mg/m^2^/day, day2–6, Mesna 600 mg/m^2^/day, day1–3 | Arm1: 29;  Arm2: 27 |
| Mike G. Martin | 2009 | American Journal of Hematology | USA | A retrospective study | - | Relapsed/refractory AML aged 18 years and older | January 1 2001 - July 31 2008 | FLAG-IDA+GO: 47 (20–68) years; FLAG-IDA: 48 (18–70) years | 71 | Induction regimen | GO 9 mg/m^2^ on Day 8 | FLAG-IDA (fludarabine 25 mg/m^2^ IV, Day1–5; Ara-C 2g/m^2^ IV, Day1–5; idarubicin 12 mg/m^2^, Day1–3) | 48 | As shown in the concomitant therapy | 23 |
| Elihu H. Estey | 2002 | Blood | USA | A retrospective study | - | Patients aged ≥ 65 years, with newly diagnosed AML, refractory anemia (RA) with excess of blasts in transformation (RAEB-t), or RA with excess blasts (RAEB), had a karyotype other than inv(16), t(8;21), or t(15;17); | IA: 1991-1992; GO: January 1st 2000 - 2002 | GO: 71 (65-89) years; IA: 72 (65-84) years | 82 | Induction and consolation regimen | 22 patients received 9 mg/m^2^ GO on  days 1 and 15. When only 5 of the 22 patients had CR, the remaining 29 patients received 9 mg/m^2^ GO on days 1 and 8. In remission, GO was at the dose of 6 mg/m^2^ on day 1. | All patients were randomized to receive or not 15 µg/kg/day IL-11 on day3-28 or not. In remission, patients received one course of GO, cyclosporine A, fludarabine, and Ara-C alternating every 5 weeks for 10 months with one course of IA. 6 mg/kg cyclosporine A followed by 16 mg/kg daily on days 1 and 2; 15 mg/m^2^ fludarabine twice daily on day2-6; and 0.5 g/m^2^ Ara-C twice daily on day2-6. IA consisted of 8 mg/m^2^ idarubicin daily on day1-2 and 1.5 g/m^2^ Ara-C daily on day1-2. | 51 | Induction: IA, 12 mg/m^2^ idarubicin daily on day1-3 and 1.5 g/m^2^ of Ara-C daily on day1-3 [continuous infusion]; Consolidation: Ara-C (100 mg/m^2^ daily for 5 days [continuous infusion]) alternating every 5 weeks for 10 months with IA as described above for the GO program. | 31 |

Abbreviations: AML, acute myeloid leukemia; NPM1, Nucleophosmin 1; GO, gemtuzumab ozogamicin; IV, intravenously; Ara-C, cytosine arabinoside (cytarabine); ICE, idarubicin + Ara-C + etoposide; ATRA, all-trans retinoic acid; PO, orally; BSC, best supportive care; allo-HSCT, allogeneic hematopoietic stem cell transplant; CBF-AML, Core binding factor acute myeloid leukemia; G-SCF, granulocyte-colony stimulating factor; FA, fludarabine + Ara-C; FLAG, fludarabine + Ara-C + G-CSF; A-HAE, High-dose Ara-C + etoposide + ATRA; IA, idarubicin + Ara-C; BID, bis in die; CR, complete remission; CRi, CR with incomplete blood count recovery; PR, partial remission; FLAG-IDA, FLAG+ idarubicin; HAM, high-dose Ara-C + mitoxantrone; A-HAE, high-dose Ara-C + etoposide + ATRA; HiDAC, high-dose Ara-C; FLAI, fludarabine + Ara-C + idarubicin; My-FLAI, FLAI + GO; MyAIE, GO + idarubicin + Ara-C + etoposide; FLAN, fludarabine + Ara-C + mitoxantrone; DAE/ADE, daunorubicin + Ara-C + etoposide; BSC, best supportive care; WBC, white blood cells; MRD, minimal residual disease; MICE, mitoxantrone + etoposide + Ara-C; ICE, idarubicin + Ara-C + etoposide; ASCT, autologous stem cell transplantation; CHT, chemotherapy; IA, idarubicin + Ara-c.

**Supplementary Table 3: NOS score of retrospective studies**

| Study | Year | Selection | | | | Comparability | Outcome | | | Score |
| --- | --- | --- | --- | --- | --- | --- | --- | --- | --- | --- |
|  |  | Representativeness of the exposed cohort | Selection of the non-exposed cohort | Ascertainment of exposure | Demonstration that outcome of interest was not present at start of study | Comparability of cohorts on the basis of the design or analysis | Assessment of outcome | Was follow-up long enough for outcomes to occur (5 years) | Adequacy of follow up of cohorts |  |
| Vincent T. Ho | 2019 | ☆ | ☆ | ☆ | ☆ | ☆ | ☆ | ☆ | ☆ | 8 |
| Gautam M. Borthakur | 2019 | ☆ | ☆ | ☆ | ☆ | - | ☆ | ☆ | ☆ | 7 |
| M Wattad | 2017 | ☆ | ☆ | ☆ | ☆ | - | ☆ | ☆ | ☆ | 7 |
| Michele Gottardi | 2017 | ☆ | ☆ | ☆ | ☆ | ☆ | ☆ | - | ☆ | 7 |
| Katherine Tarlock | 2016 | ☆ | ☆ | ☆ | ☆ | ☆ | ☆ | ☆ | ☆ | 8 |
| Sartor, Chiara | 2016 | ☆ | ☆ | ☆ | ☆ | - | ☆ | ☆ | - | 6 |
| Juan M. Bergua | 2016 | ☆ | ☆ | ☆ | ☆ | ☆ | ☆ | ☆ | ☆ | 8 |
| Jurjen Versluis | 2015 | ☆ | ☆ | ☆ | ☆ | - | ☆ | ☆ | ☆ | 7 |
| Marie-Anne Hospital | 2014 | ☆ | ☆ | ☆ | ☆ | ☆ | ☆ | - | ☆ | 7 |
| Marino Clavio | 2013 | ☆ | ☆ | ☆ | ☆ | ☆ | ☆ | - | ☆ | 7 |
| Thomas Prebet | 2011 | ☆ | ☆ | ☆ | ☆ | ☆ | ☆ | - | ☆ | 7 |
| Antonella Poloni | 2010 | ☆ | ☆ | ☆ | ☆ | ☆ | ☆ | ☆ | ☆ | 8 |
| Mike G. Martin | 2009 | ☆ | ☆ | ☆ | ☆ | - | ☆ | - | ☆ | 6 |
| Elihu H. Estey | 2002 | ☆ | ☆ | ☆ | ☆ | - | ☆ | - | ☆ | 6 |

**Supplementary Table 4: Pooled RR and 95% CI of CR before and after sensitivity analyses**

|  |  | **Results before sensitive analyses** | | | | | | | | **Results after sensitive analyses** | | | | | | | | **Source of heterogeneity** | | |
| --- | --- | --- | --- | --- | --- | --- | --- | --- | --- | --- | --- | --- | --- | --- | --- | --- | --- | --- | --- | --- |
|  | **Groups** | **GO** | **Control** | **Pooled RR** | **Lower 95%CI** | **Upper 95%CI** | ***p*** | ***I^2^*** | ***p* of heterogeneity** | **GO** | **Control** | **pooled RR** | **Lower 95%CI** | **Upper 95%CI** | ***p*** | ***I^2^*** | ***p* of heterogeneity** | **Study** | **Reason** |  |
| Subgroup | The whole group | 2487/3392 | 2791/4326 | 0.95 | 0.89 | 1 | 0.084 | 63.6% | 0.000 |  |  |  |  |  |  |  |  |  | Unstable model |  |
| Karyotypes | Low / intermediate risk | 41/62 | 40/61 | 1.01 | 0.78 | 1.3 | 0.939 | - | - |  |  |  |  |  |  |  |  |  |  |  |
|  | Low risk | 253/268 | 252/275 | 0.99 | 0.85 | 1.14 | 0.893 | 42.8% | 0.120 |  |  |  |  |  |  |  |  |  |  |  |
|  | Intermediate risk | 1011/1191 | 983/1219 | 0.94 | 0.89 | 0.99 | **0.023** | 9.1% | 0.358 |  |  |  |  |  |  |  |  |  |  |  |
|  | High risk | 179/330 | 182/339 | 1.03 | 0.84 | 1.25 | 0.771 | 0.0% | 0.954 |  |  |  |  |  |  |  |  |  |  |  |
| NPM1 mutation | + | 353/401 | 357/402 | 0.88 | 0.63 | 1.22 | 0.448 | 38.2% | 0.183 |  |  |  |  |  |  |  |  |  |  |  |
|  | - | 198/259 | 227/314 | 0.79 | 0.58 | 1.07 | 0.131 | 0.0% | 0.998 |  |  |  |  |  |  |  |  |  |  |  |
| FLT3-ITD mutation | + | 86/104 | 98/120 | 0.83 | 0.51 | 1.35 | 0.453 | 0.0% | 0.979 |  |  |  |  |  |  |  |  |  |  |  |
|  | - | 275/348 | 258/350 | 0.7 | 0.51 | 0.97 | **0.030** | 0.0% | 0.907 |  |  |  |  |  |  |  |  |  |  |  |
| Age | <60 years old | 1179/1381 | 1159/1392 | 0.97 | 0.93 | 1 | 0.100 | 0.0% | 0.643 |  |  |  |  |  |  |  |  |  |  |  |
|  | ≥60 years old | 596/941 | 606/953 | 0.95 | 0.79 | 1.15 | 0.592 | 22.3% | 0.260 |  |  |  |  |  |  |  |  |  |  |  |
| Age | <70 years old | 1660/2110 | 1625/2129 | 0.97 | 0.92 | 1.02 | 0.247 | 33.9% | 0.127 |  |  |  |  |  |  |  |  |  |  |  |
|  | ≥70 years old | 142/254 | 154/253 | 0.77 | 0.45 | 1.31 | 0.338 | 37.2% | 0.204 |  |  |  |  |  |  |  |  |  |  |  |
| Gender | Male | 440/554 | 432/563 | 0.88 | 0.72 | 1.08 | 0.217 | 0.0% | 0.963 |  |  |  |  |  |  |  |  |  |  |  |
|  | Female | 474/640 | 480/602 | 1.09 | 0.78 | 1.54 | 0.619 | 49.4% | 0.139 |  |  |  |  |  |  |  |  |  |  |  |
| CD33 | CD33+ | 685/889 | 663/893 | 0.9 | 0.76 | 1.08 | 0.240 | 2.8% | 0.358 |  |  |  |  |  |  |  |  |  |  |  |
|  | CD33- | 71/98 | 64/89 | 1.01 | 0.53 | 1.93 | 0.976 | 0.0% | 0.452 |  |  |  |  |  |  |  |  |  |  |  |
| AML type | de novo AML | 1333/1607 | 1307/1630 | 0.96 | 0.91 | 1 | 0.090 | 0.0% | 0.599 |  |  |  |  |  |  |  |  |  |  |  |
|  | secondary AML | 92/166 | 109/173 | 1.31 | 0.91 | 1.89 | 0.148 | 0.0% | 0.828 |  |  |  |  |  |  |  |  |  |  |  |
| Dose of GO | GO<6mg/m^2^ | 1853/2564 | 2019/2649 | 0.97 | 0.92 | 1.02 | 0.247 | 44.9% | 0.069 | 1740/2162 | 1829/2379 | 1 | 0.96 | 1.03 | 1 | 11.9% | 0.338 | Chiara 2016 | Untreated CD33+ non-M3 AML patients aged ≥60. |  |
|  | GO≥6mg/m^2^ | 564/951 | 528/942 | 0.96 | 0.82 | 1.12 | 0.608 | 68.7% | 0.002 |  |  |  |  |  |  |  |  |  | Unstable model |  |
| Combination regimens | GO+DA | 1048/1349 | 1012/1353 | 0.96 | 0.92 | 1.01 | 0.086 | 0.0% | 0.742 |  |  |  |  |  |  |  |  |  |  |  |
|  | GO+FLAG | 423/556 | 590/870 | 0.92 | 0.71 | 1.19 | 0.527 | 76.3% | 0.001 | 310/417 | 400/600 | 0.8 | 0.68 | 0.95 | **0.009** | 30.6% | 0.218 | Chiara 2016 | Untreated CD33+  non-M3 AML patients aged ≥60. |  |
|  | GO alone | 111/302 | 259/885 | 0.5 | 0.11 | 2.33 | 0.373 | 90.8% | 0.000 |  |  |  |  |  |  |  |  |  | Unstable model |  |
|  | GO + others | 905/1185 | 929/1217 | 0.99 | 0.89 | 1.1 | 0.852 | 65.4% | 0.013 |  |  |  |  |  |  |  |  |  | Unstable model |  |

Abbreviations: CR, complete remission; GO, gemtuzumab ozogamicin; RR, relative risk; 95% CI, 95% confidence Interval; AML, acute myeloid leukemia; NPM1, Nucleophosmin 1; DA, daunorubicin and cytarabine; FLAG, fludarabine + cytarabine + granulocyte-colony stimulating factor; FLT3-ITD, FMS-like tyrosine kinase 3 internal tandem duplication. P-value in bold meant the statistically significant.

**Supplementary Table 5. Pooled HR and 95% CI of OS before and after sensitivity analyses**

|  |  | **Results before sensitive analyses** | | | | | | | | **Results after sensitive analyses** | | | | | | | | **Source of heterogeneity** | |
| --- | --- | --- | --- | --- | --- | --- | --- | --- | --- | --- | --- | --- | --- | --- | --- | --- | --- | --- | --- |
|  | **Groups** | **GO** | **Control** | **pooled HR** | **Lower 95%CI** | **Upper 95%CI** | ***p*** | ***I^2^*** | ***p* of heterogeneity** | **GO** | **Control** | **pooled HR** | **Lower 95%CI** | **Upper 95%CI** | ***p*** | ***I^2^*** | ***p* of heterogeneity** | **Study** | **Reason** |
|  | The whole group | 3536 | 4553 | 0.86 | 0.78 | 0.95 | **0.003** | 62.1% | 0.000 |  |  |  |  |  |  |  |  |  | Stable model |
| Karyotypes | Low / intermediate risk | 302 | 299 | 0.69 | 0.43 | 1.11 | 0.125 | 70.4% | 0.018 | 215 | 203 | 0.55 | 0.4 | 0.74 | **0.000** | 0.0% | 0.908 | Amadori 2013 | Different therapies from other studies: MICE: mitoxantrone + etoposide + Ara-C; ICE: idarubicin + Ara-C + etoposide. |
|  | Low risk | 318 | 373 | 0.62 | 0.45 | 0.86 | **0.004** | 48.3% | 0.072 | 246 | 308 | 0.72 | 0.58 | 0.9 | **0.003** | 3.7% | 0.393 | Burnett 2011 | Unknown reasons. This cohort overlapped with other studies in age, doses of GO, strategy, and AML subtype. |
|  | Intermediate risk | 1278 | 1303 | 0.91 | 0.87 | 0.96 | **0.000** | 0.0% | 0.459 |  |  |  |  |  |  |  |  |  |  |
|  | High risk | 522 | 578 | 0.95 | 0.87 | 1.03 | 0.234 | 1.4% | 0.426 |  |  |  |  |  |  |  |  |  |  |
| NPM1 mutation | + | 144 | 143 | 0.67 | 0.47 | 0.95 | **0.026** | 0.0% | 0.670 |  |  |  |  |  |  |  |  |  |  |
|  | - | 326 | 381 | 0.81 | 0.67 | 0.99 | **0.034** | 0.0% | 0.980 |  |  |  |  |  |  |  |  |  |  |
| FLT3-ITD mutation | + | 183 | 168 | 0.88 | 0.62 | 1.26 | 0.480 | 23.8% | 0.268 |  |  |  |  |  |  |  |  |  |  |
|  | - | 420 | 437 | 0.77 | 0.64 | 0.93 | **0.006** | 0.0% | 0.759 |  |  |  |  |  |  |  |  |  |  |
| Age | <60 years old | 1633 | 1651 | 0.93 | 0.79 | 1.09 | 0.377 | 41.7% | 0.057 | 1625 | 1645 | 0.94 | 0.81 | 1.09 | 0.414 | 35.6% | 0.105 | Burnett 2011, 0-14 years | Age: 0-14, GO: 8, non-GO: 6; Less cases than other researches. |
|  | ≥60 years old | 1445 | 1660 | 0.87 | 0.76 | 0.99 | **0.039** | 47.2% | 0.03 | 1209 | 1424 | 0.83 | 0.75 | 0.92 | **0.000** | 10.8% | 0.339 | Amadori 2013 | Different therapies from other studies: MICE: mitoxantrone + etoposide + Ara-C; ICE: idarubicin + Ara-C + etoposide. |
| Age | <70 years old | 2472 | 2500 | 0.9 | 0.82 | 1 | **0.037** | 37.3% | 0.066 | 2438 | 2444 | 0.92 | 0.85 | 1 | **0.044** | 18.9% | 0.242 | Prebet 2011 | Different therapies from other studies: Ara-C >500 mg/m^2^ alone; MEC: Ara-C 1000 mg/m^2^/d, day1-5; Mitoxantrone 8 mg/m^2^/d, day1-5; Etoposide 100 mg/m^2^/d, day1-5; Ara-C 2g/m^2^/d, day 1-5, Etoposide 100 mg/m^2^/d, day1-5; And Ara-C 2g/m^2^/d, day1-5, Topotecan 1.5 mg/m^2^/d, day1-5; Others. |
|  | ≥70 years old | 331 | 327 | 0.9 | 0.64 | 1.26 | 0.542 | 68.6% | 0.013 | 248 | 243 | 0.79 | 0.62 | 1 | 0.053 | 23.4% | 0.27 | Amadori 2013 | Different therapies from other studies: MICE: mitoxantrone + etoposide + Ara-C; ICE: idarubicin + Ara-C + etoposide. |
| Gender | Male | 681 | 712 | 0.85 | 0.75 | 0.96 | **0.010** | 0.0% | 0.85 |  |  |  |  |  |  |  |  |  |  |
|  | Female | 550 | 518 | 0.83 | 0.59 | 1.17 | 0.286 | 77.4% | 0.012 | 489 | 472 | 0.97 | 0.79 | 1.20 | 0.775 | 37.3% | 0.207 | Amadori 2016 | This included all patients were over the age of 75 years, as well as aged from 61 to 75 years old, with a WHO performance score greater than 2 or who were unwilling to receive standard chemotherapy. |
| CD33 | CD33+ | 1182 | 1169 | 0.83 | 0.69 | 1.01 | **0.055** | 60.7% | 0.018 | 1134 | 1122 | 0.89 | 0.76 | 1.05 | 0.158 | 42.2% | 0.124 | Amadori 2016 with >80% CD33+ | The expression of CD33 in this group was the highest. |
|  | CD33- | 136 | 132 | 1.06 | 0.79 | 1.42 | 0.697 | 0.0% | 0.903 |  |  |  |  |  |  |  |  |  |  |
| AML type | de novo AML | 2033 | 2052 | 0.90 | 0.79 | 1.02 | 0.106 | 47.3% | 0.056 | 1868 | 1877 | 0.86 | 0.79 | 0.94 | **0.001** | 0.0% | 0.462 | Amadori 2013 | Different therapies from other studies: MICE: mitoxantrone + etoposide + Ara-C; ICE: idarubicin + Ara-C + etoposide. |
|  | secondary AML | 249 | 237 | 0.9 | 0.69 | 1.16 | 0.427 | 32.7% | 0.216 |  |  |  |  |  |  |  |  |  |  |
| Treatment stage | Induction with/without consolidation regimen | 3091 | 3882 | 0.86 | 0.76 | 0.96 | **0.011** | 65.9% | 0.000 |  |  |  |  |  |  |  |  |  | Stable model |
|  | Consolidation regimen | 912 | 1138 | 0.92 | 0.75 | 1.13 | 0.425 | 45.6% | 0.101 |  |  |  |  |  |  |  |  |  |  |
| Dose of GO | GO<6mg/m^2^ | 2220 | 2958 | 0.82 | 0.71 | 0.95 | **0.008** | 55.1% | 0.011 | 2081 | 2688 | 0.87 | 0.77 | 0.99 | **0.030** | 36.1% | 0.11 | Chiara 2016 | Unknown reasons. This cohort overlapped with other studies in age, GO doses, strategy, and AML subtype. |
|  | GO≥6mg/m^2^ | 1316 | 1595 | 0.9 | 0.77 | 1.05 | 0.183 | 66.3% | 0.001 |  |  |  |  |  |  |  |  |  | Stable model |
| Combination regimens | GO+DA | 1487 | 1486 | 0.93 | 0.8 | 1.07 | 0.328 | 47.2% | 0.092 | 1192 | 1186 | 0.88 | 0.78 | 0.98 | **0.028** | 6.6% | 0.369 | Petersdorf 2013 | Unknown reasons. This cohort overlapped with others in age, GO doses, strategy, and AML subtype. |
|  | GO+FLAG | 538 | 837 | 0.8 | 0.63 | 1 | 0.058 | 70.4% | 0.005 | 399 | 567 | 0.91 | 0.78 | 1.07 | 0.242 | 29.2% | 0.227 | Chiara 2016 | Unknown reasons. This cohort overlapped with other studies in age, doses of GO, strategy, and AML subtype. |
|  | GO alone | 366 | 597 | 0.74 | 0.61 | 0.91 | **0.003** | 30.2% | 0.231 |  |  |  |  |  |  |  |  |  |  |
|  | GO + others | 1190 | 1679 | 0.93 | 0.75 | 1.15 | 0.506 | 58.1% | 0.014 | 1156 | 1623 | 1.02 | 0.86 | 1.2 | 0.816 | 35.1% | 0.148 | Prebet 2011 | The regimens of this study were different from other studies. |

Abbreviations: OS, overall survival; GO, gemtuzumab ozogamicin; HR, hazard ratio; 95% CI, 95% confidence Interval; AML, acute myeloid leukemia; NPM1, Nucleophosmin 1; DA, daunorubicin and cytarabine; FLAG, fludarabine + cytarabine + granulocyte-colony stimulating factor; Ara-C, cytosine arabinoside (cytarabine); FLT3-ITD, FMS-like tyrosine kinase 3 internal tandem duplication. P-value in bold meant the statistically significant.

**Supplementary Table 6. Pooled HR and 95% CI of EFS before and after sensitivity analyses**

|  |  | **Results before sensitive analyses** | | | | | | | | **Results after sensitive analyses** | | | | | | | | **Source of heterogeneity** | |
| --- | --- | --- | --- | --- | --- | --- | --- | --- | --- | --- | --- | --- | --- | --- | --- | --- | --- | --- | --- |
|  | **Groups** | **GO** | **Control** | **pooled HR** | **Lower 95%CI** | **Upper 95%CI** | ***p*** | ***I^2^*** | ***p* of heterogeneity** | **GO** | **Control** | **pooled HR** | **Lower 95%CI** | **Upper 95%CI** | ***p*** | ***I^2^*** | ***p* of heterogeneity** | **Study** | **Reason** |
|  | The whole group | 1415 | 1489 | 0.86 | 0.76 | 0.97 | **0.015** | 62.4% | 0.003 |  |  |  |  |  |  |  |  |  | Unstable model |
| Karyotypes | Low / intermediate risk | 574 | 577 | 0.81 | 0.69 | 0.95 | **0.010** | 32.7% | 0.204 |  |  |  |  |  |  |  |  |  |  |
|  | High risk | 153 | 216 | 0.83 | 0.68 | 1.02 | 0.065 | 15.7% | 0.305 |  |  |  |  |  |  |  |  |  |  |
| NPM1 mutation | + | 337 | 344 | 0.69 | 0.41 | 1.16 | 0.162 | 54.5% | 0.138 |  |  |  |  |  |  |  |  |  |  |
|  | - | 91 | 90 | 0.64 | 0.4 | 1.03 | 0.064 | - | - |  |  |  |  |  |  |  |  |  | Only one study |
| FLT3-ITD mutation | + | 168 | 132 | 0.80 | 0.37 | 1.71 | 0.568 | 84.3% | 0.002 | 146 | 105 | 1.16 | 0.7 | 1.93 | 0.566 | 61.4% | 0.108 | Castaigne 2014 | “7+3” regimen was used for older patients, which was different from other studies. |
|  | - | 357 | 358 | 0.70 | 0.56 | 0.88 | **0.002** | 0.0% | 0.670 |  |  |  |  |  |  |  |  |  |  |
| Age | <60 years old | 592 | 611 | 0.84 | 0.65 | 1.1 | 0.194 | 28.7% | 0.240 |  |  |  |  |  |  |  |  |  |  |
|  | ≥60 years old | 442 | 430 | 0.94 | 0.75 | 1.19 | 0.599 | 57.6% | 0.070 | 342 | 344 | 1.02 | 0.89 | 1.17 | 0.777 | 0.0% | 0.380 | Castaigne 2014 | “7+3” regimen was used for older patients, which was different from other studies. |
| Gender | Male | 203 | 204 | 0.8 | 0.43 | 1.49 | 0.482 | 80.8% | 0.022 |  |  |  |  |  |  |  |  |  | Only two studies |
|  | Female | 224 | 228 | 0.63 | 0.49 | 0.81 | **0.000** | 0.0% | 0.477 |  |  |  |  |  |  |  |  |  |  |
| AML type | de novo AML | 876 | 894 | 0.80 | 0.68 | 0.94 | **0.007** | 61.3% | 0.052 | 737 | 755 | 0.87 | 0.79 | 0.95 | **0.003** | 0.0% | 0.501 | Castaigne 2014 | “7+3” regimen was used for older patients, which was different from other studies. |
|  | secondary AML | 33 | 30 | 0.74 | 0.37 | 1.47 | 0.392 | - | - |  |  |  |  |  |  |  |  |  | Only one study |
| Treatment stage | Induction with / without consolidation regimen | 1356 | 1428 | 0.86 | 0.75 | 0.98 | **0.027** | 66.1% | 0.002 |  |  |  |  |  |  |  |  |  | Unstable model |
|  | Consolidation regimen | 59 | 61 | 0.82 | 0.39 | 1.72 | 0.600 | - | - |  |  |  |  |  |  |  |  |  | Only one study |
| Dose of GO | GO<6mg/m^2^ | 1040 | 1116 | 0.81 | 0.72 | 0.92 | **0.001** | 47.6% | 0.075 | 901 | 977 | 0.85 | 0.77 | 0.93 | **0.001** | 10.9% | 0.346 | Castaigne 2014 | “7+3” regimen was used for older patients, which was different from other studies. |
|  | GO≥6mg/m^2^ | 375 | 373 | 0.95 | 0.7 | 1.27 | 0.736 | 69.6% | 0.02 | 341 | 317 | 1.04 | 0.87 | 1.25 | 0.671 | 32.8% | 0.226 | Prebet 2011 | This study included Ara-C >500 mg/m^2^; MEC: Ara-C 1 g/m^2^/d, day1-5; Mitoxantrone 8 mg/m^2^/d, day1-5; Etoposide 100 mg/m^2^/d, day1-5; Ara-C 2g/m^2^/d, day1-5, Etoposide 100 mg/m^2^/d, day1-5; Ara-C 2g/m^2^/d, day1-5, Topotecan 1.5 mg/m^2^/d, day1-5; Others, which were different from other studies. |

Abbreviations: EFS, event-free survival; GO, gemtuzumab ozogamicin; HR, hazard ratio; 95% CI, 95% confidence Interval; AML, acute myeloid leukemia; NPM1, Nucleophosmin 1; FLT3-ITD, FMS-like tyrosine kinase 3 internal tandem duplication. P-value in bold meant the statistically significant.

**Supplementary Table 7. Pooled HR and 95% CI of RFS before and after sensitivity analyses**

|  |  | **Results before sensitive analyses** | | | | | | | | **Results after sensitive analyses** | | | | | | | | **Source of heterogeneity** | | |
| --- | --- | --- | --- | --- | --- | --- | --- | --- | --- | --- | --- | --- | --- | --- | --- | --- | --- | --- | --- | --- |
|  | **Groups** | **GO** | **Control** | **pooled HR** | **Lower 95%CI** | **Upper 95%CI** | ***p*** | ***I^2^*** | ***p* of heterogeneity** | **GO** | **Control** | **pooled HR** | **Lower 95%CI** | **Upper 95%CI** | ***p*** | ***I^2^*** | ***p* of heterogeneity** | **Study** | **Reason** |  |
|  | The whole group | 2490 | 3260 | 0.83 | 0.74 | 0.93 | **0.001** | 59.2% | 0.001 |  |  |  |  |  |  |  |  |  | Stable model |  |
| Karyotypes | Low / intermediate risk | 127 | 120 | 0.58 | 0.42 | 0.8 | **0.001** | 0.0% | 0.580 |  |  |  |  |  |  |  |  |  |  |  |
|  | Low risk | 330 | 425 | 0.62 | 0.45 | 0.86 | **0.004** | 41.2% | 0.130 |  |  |  |  |  |  |  |  |  |  |  |
|  | Intermediate risk | 651 | 648 | 0.95 | 0.87 | 1.03 | 0.234 | 18.5% | 0.293 |  |  |  |  |  |  |  |  |  |  |  |
|  | High risk | 134 | 130 | 0.91 | 0.75 | 1.1 | 0.334 | 0.0% | 0.543 |  |  |  |  |  |  |  |  |  |  |  |
| NPM1 mutation | + | 106 | 106 | 0.65 | 0.43 | 0.98 | **0.040** | 0.0% | 0.326 |  |  |  |  |  |  |  |  |  |  |  |
|  | - | 180 | 198 | 0.82 | 0.58 | 1.15 | 0.256 | 19.2% | 0.266 |  |  |  |  |  |  |  |  |  |  |  |
| FLT3-ITD mutation | + | 125 | 118 | 0.74 | 0.46 | 1.18 | 0.210 | 40.4% | 0.187 |  |  |  |  |  |  |  |  |  |  |  |
|  | - | 255 | 238 | 0.78 | 0.55 | 1.12 | 0.171 | 36.5% | 0.209 |  |  |  |  |  |  |  |  |  |  |  |
| Age | <60 years old | 1252 | 1242 | 0.9 | 0.8 | 1 | 0.064 | 0.0% | 0.503 |  |  |  |  |  |  |  |  |  |  |  |
|  | ≥60 years old | 563 | 828 | 0.79 | 0.68 | 0.93 | **0.003** | 40.0% | 0.112 |  |  |  |  |  |  |  |  |  |  |  |
| CD33 | CD33+ | 401 | 403 | 0.75 | 0.59 | 0.95 | **0.018** | 29.7% | 0.233 |  |  |  |  |  |  |  |  |  |  |  |
|  | CD33- | 37 | 27 | 1.14 | 0.62 | 2.09 | 0.673 | - | - |  |  |  |  |  |  |  |  |  | Only one study |  |
| AML type | de novo AML | 1104 | 1086 | 0.82 | 0.69 | 0.98 | **0.027** | 55.9% | 0.059 | 991 | 982 | 0.87 | 0.78 | 0.98 | **0.017** | 0.0% | 0.545 | Castaigne 2014 | Unknown reasons. This cohort overlapped with others in age, GO doses, strategy, and AML subtype. |  |
|  | secondary AML | 30 | 36 | 0.84 | 0.48 | 1.47 | 0.541 | - | - |  |  |  |  |  |  |  |  |  | Only one study |  |
| Treatment stage | Induction with / without consolidation regimen | 2044 | 2559 | 0.8 | 0.69 | 0.93 | **0.003** | 67.5% | 0.000 |  |  |  |  |  |  |  |  |  | Stable model |  |
|  | Consolidation regimen | 564 | 810 | 0.93 | 0.75 | 1.15 | 0.506 | 50.1% | 0.061 |  |  |  |  |  |  |  |  |  | Stable model |  |
| Dose of GO | GO<6mg/m^2^ | 1570 | 1593 | 0.76 | 0.65 | 0.87 | **0.000** | 45.9% | 0.064 | 1513 | 1488 | 0.78 | 0.68 | 0.88 | **0.000** | 35.3% | 0.147 | Borthakur 2019 | Unknown reasons. This cohort overlapped with others in age, doses of GO, strategy, and AML subtype. |  |
|  | GO≥6mg/m^2^ | 920 | 1667 | 0.91 | 0.77 | 1.08 | 0.275 | 59.4% | 0.012 |  |  |  |  |  |  |  |  |  | Stable model |  |
| Combination regimens | GO+DA | 1056 | 1027 | 0.84 | 0.69 | 1.01 | 0.073 | 60.8% | 0.037 | 943 | 923 | 0.89 | 0.79 | 1 | 0.053 | 0.0% | 0.414 | Castaigne 2014 | Unknown reasons. This cohort overlapped with others in age, doses of GO, strategy, and AML subtype. |  |
|  | GO+FLAG | 312 | 371 | 0.74 | 0.56 | 0.99 | **0.038** | 50.3% | 0.110 |  |  |  |  |  |  |  |  |  |  |  |
|  | GO alone | 312 | 580 | 0.86 | 0.73 | 1.02 | 0.077 | 3.3% | 0.376 |  |  |  |  |  |  |  |  |  |  |  |
|  | GO + others | 810 | 1282 | 0.85 | 0.67 | 1.09 | 0.191 | 70.6% | 0.002 |  |  |  |  |  |  |  |  |  | Stable model |  |

Abbreviations: RFS, relapse-free survival; GO, gemtuzumab ozogamicin; HR, hazard ratio; 95% CI, 95% confidence Interval; AML, acute myeloid leukemia; NPM1, Nucleophosmin 1; DA, daunorubicin and cytarabine; FLAG, fludarabine + cytarabine + granulocyte-colony stimulating factor; Ara-C, cytosine arabinoside (cytarabine); FLT3-ITD, FMS-like tyrosine kinase 3 internal tandem duplication. P-value in bold meant the statistically significant.

**Supplementary Table 8. Pooled HR and 95% CI of CIR before and after sensitivity analyses**

|  |  | **Results before sensitive analyses** | | | | | | | | **Results after sensitive analyses** | | | | | | | | **Source of heterogeneity** | | |
| --- | --- | --- | --- | --- | --- | --- | --- | --- | --- | --- | --- | --- | --- | --- | --- | --- | --- | --- | --- | --- |
|  | **Groups** | **GO** | **Control** | **pooled HR** | **Lower 95%CI** | **Upper 95%CI** | ***p*** | ***I^2^*** | ***p* of heterogeneity** | **GO** | **Control** | **pooled HR** | **Lower 95%CI** | **Upper 95%CI** | ***p*** | ***I^2^*** | ***p* of heterogeneity** | **Study** | **Reason** |  |
|  | The whole group | 2560 | 3218 | 0.86 | 0.76 | 0.98 | **0.020** | 58.2% | 0.003 | 2423 | 2670 | 0.82 | 0.74 | 0.90 | **0.000** | 21.8% | 0.223 | Ho 2019 | GO was 9.5 (3.0-33.0) mg/m^2^, which was higher than other studies. The combination regimen of GO was not clear in this study. |  |
| Karyotypes | Low/intermediate risk | 96 | 99 | 0.95 | 0.37 | 1.21 | 0.865 | - | - |  |  |  |  |  |  |  |  |  | Only one study |  |
|  | Low risk | 208 | 186 | 0.71 | 0.44 | 1.15 | 0.162 | 28.6% | 0.246 |  |  |  |  |  |  |  |  |  |  |  |
|  | Intermediate risk | 893 | 888 | 0.81 | 0.70 | 0.93 | **0.004** | 0.0% | 0.971 |  |  |  |  |  |  |  |  |  |  |  |
|  | High risk | 213 | 225 | 0.73 | 0.53 | 1.01 | 0.056 | 11.9% | 0.333 |  |  |  |  |  |  |  |  |  |  |  |
| NPM1 mutation | + | 396 | 409 | 0.64 | 0.51 | 0.81 | **0.000** | 0.0% | 0.737 |  |  |  |  |  |  |  |  |  |  |  |
|  | - | 307 | 349 | 0.78 | 0.61 | 1.00 | **0.049** | 0.0% | 0.444 |  |  |  |  |  |  |  |  |  |  |  |
| FLT3-ITD mutation | + | 179 | 185 | 0.72 | 0.49 | 1.06 | 0.095 | 32.0% | 0.208 |  |  |  |  |  |  |  |  |  |  |  |
|  | - | 616 | 628 | 0.70 | 0.56 | 0.87 | **0.002** | 23.8% | 0.268 |  |  |  |  |  |  |  |  |  |  |  |
| Age | <60 years old | 1408 | 1388 | 0.83 | 0.73 | 0.93 | **0.003** | 0.0% | 0.467 |  |  |  |  |  |  |  |  |  |  |  |
|  | ≥60 years old | 902 | 1178 | 0.83 | 0.74 | 0.93 | **0.001** | 0.0% | 0.487 |  |  |  |  |  |  |  |  |  |  |  |
| Age | <70 years old | 1805 | 1787 | 0.80 | 0.73 | 0.89 | **0.000** | 0.0% | 0.576 |  |  |  |  |  |  |  |  |  |  |  |
|  | ≥70 years old | 264 | 256 | 0.80 | 0.64 | 1.00 | 0.050 | 0.0% | 0.544 |  |  |  |  |  |  |  |  |  |  |  |
| Gender | Male | 583 | 604 | 0.78 | 0.66 | 0.91 | **0.002** | 0.0% | 0.669 |  |  |  |  |  |  |  |  |  |  |  |
|  | Female | 536 | 522 | 0.76 | 0.54 | 1.06 | 0.111 | 69.5% | 0.038 | 390 | 384 | 0.89 | 0.73 | 1.09 | 0.254 | 0.0% | 0.700 | Schlenk 2019 | This is the only study limited to the NPM1 (+) AML. |  |
| CD33 | CD33+ | 673 | 649 | 0.75 | 0.59 | 0.96 | **0.021** | 51.2% | 0.129 |  |  |  |  |  |  |  |  |  |  |  |
|  | CD33- | 73 | 66 | 0.90 | 0.59 | 1.38 | 0.627 | 0.0% | 0.560 |  |  |  |  |  |  |  |  |  |  |  |
| AML type | de novo AML | 1616 | 1584 | 0.77 | 0.69 | 0.87 | **0.000** | 17.1% | 0.303 |  |  |  |  |  |  |  |  |  |  |  |
|  | secondary AML | 109 | 118 | 1.04 | 0.72 | 1.50 | 0.834 | 0.0% | 0.418 |  |  |  |  |  |  |  |  |  |  |  |
| Treatment stage | Induction with/without consolidation regimen | 2140 | 2540 | 0.84 | 0.71 | 1.000 | **0.050** | 69.5% | 0.001 | 2003 | 1992 | 0.79 | 0.69 | 0.89 | **0.000** | 35.5% | 0.134 | Ho 2019 | GO was 9.5 (3.0-33.0) mg/m^2^, which was higher than other studies. The combination regimen of GO was not clear in this study. |  |
|  | Consolidation regimen | 1005 | 1254 | 0.91 | 0.74 | 1.12 | 0.372 | 58% | 0.027 | 972 | 1229 | 0.95 | 0.82 | 1.09 | 0.480 | 22.7% | 0.264 | Tarlock 2016 | The cohort was so young (Median age: GO: 12.1 (1.6–20.9) years; non-GO: 13.2 (0.7–20.4) years). Although Halse 2012 also included pediatric patients, all of their patients belonged to the high-risk stratification. |  |
| Dose of GO | GO<6mg/m^2^ | 1741 | 1730 | 0.76 | 0.67 | 0.85 | **0.000** | 17.4% | 0.293 |  |  |  |  |  |  |  |  |  |  |  |
|  | GO≥6mg/m^2^ | 819 | 1488 | 1.02 | 0.86 | 1.22 | 0.824 | 46.6% | 0.096 | 682 | 940 | 0.93 | 0.81 | 1.08 | 0.323 | 0.0% | 0.843 | Ho 2019 | GO was 9.5 (3.0-33.0) mg/m^2^, which was higher than other studies. The combination regimen of GO was not clear in this study. |  |
| Combination regimens | GO+DA | 1065 | 1024 | 0.82 | 0.73 | 0.92 | **0.001** | 0.0% | 0.441 |  |  |  |  |  |  |  |  |  |  |  |
|  | GO + others | 1495 | 2194 | 0.88 | 0.74 | 1.06 | 0.163 | 64.1% | 0.002 | 1358 | 1646 | 0.82 | 0.72 | 0.95 | **0.005** | 31.9% | 0.153 | Ho 2019 | GO was 9.5 (3.0-33.0) mg/m^2^, which was higher than other studies. The combination regimen of GO was not clear in this study. |  |

Abbreviations: CIR, cumulative incidence of risk; GO, gemtuzumab ozogamicin; HR, hazard ratio; 95% CI, 95% confidence Interval; AML, acute myeloid leukemia; NPM1, Nucleophosmin 1; DA, daunorubicin and cytarabine; FLAG, fludarabine + cytarabine + granulocyte-colony stimulating factor; Ara-C, cytosine arabinoside (cytarabine); FLT3-ITD, FMS-like tyrosine kinase 3 internal tandem duplication. P-value in bold meant the statistically significant.

**Supplementary Table 9: Pooled risk ratios of complete remission rate**

|  | **Control** | **GO_ADE** | **GO_alone** | **GO_Arac** | **GO_DA** | **GO_FLAG** | **GO_ICE_ATRA** | **GO_MICE** |
| --- | --- | --- | --- | --- | --- | --- | --- | --- |
| Control | Control | 0.99 (0.83, 1.52) | 0.02 (0, 0.25) | 1.04 (0.69, 1.57) | 0.95 (0.77, 1.08) | 1 (0.73, 1.38) | 1.04 (0.77, 1.41) | 0.86 (0.54, 1.35) |
| GO_ADE | 1.01 (0.66, 1.2) | GO_ADE | 0.02 (0, 0.25) | 1.04 (0.57, 1.58) | 0.96 (0.57, 1.16) | 1 (0.6, 1.41) | 1.05 (0.61, 1.42) | 0.85 (0.46, 1.36) |
| GO_alone | 64.46 (3.98, 1050.05) | 65.06 (4.05, 1102.09) | GO_alone | 66.91 (3.94, 1133.99) | 60.2 (3.68, 990.23) | 64.52 (3.92, 1092.48) | 67.1 (4.05, 1116.23) | 55.2 (3.31, 930.21) |
| GO_Arac | 0.96 (0.64, 1.45) | 0.96 (0.63, 1.76) | 0.01 (0, 0.25) | GO_Arac | 0.9 (0.56, 1.38) | 0.95 (0.58, 1.63) | 1 (0.6, 1.65) | 0.82 (0.44, 1.52) |
| GO_DA | 1.06 (0.93, 1.3) | 1.04 (0.86, 1.75) | 0.02 (0, 0.27) | 1.11 (0.73, 1.77) | GO_DA | 1.06 (0.76, 1.58) | 1.09 (0.81, 1.63) | 0.91 (0.57, 1.51) |
| GO_FLAG | 1 (0.72, 1.37) | 1 (0.71, 1.67) | 0.02 (0, 0.26) | 1.05 (0.62, 1.73) | 0.95 (0.63, 1.31) | GO_FLAG | 1.04 (0.67, 1.58) | 0.86 (0.49, 1.48) |
| GO_ICE_ATRA | 0.96 (0.71, 1.3) | 0.95 (0.7, 1.65) | 0.01 (0, 0.25) | 1 (0.6, 1.65) | 0.91 (0.62, 1.24) | 0.96 (0.63, 1.49) | GO_ICE_ATRA | 0.83 (0.48, 1.42) |
| GO_MICE | 1.17 (0.74, 1.84) | 1.17 (0.73, 2.18) | 0.02 (0, 0.3) | 1.22 (0.66, 2.25) | 1.1 (0.66, 1.74) | 1.16 (0.67, 2.04) | 1.21 (0.71, 2.08) | GO_MICE |

Abbreviations: GO, gemtuzumab ozogamicin; Arac, cytosine arabinoside (cytarabine); ADE, daunorubicin + Ara-C + etoposide; DA, daunorubicin + Ara-C; FLAG, fludarabine + Ara-C + granulocyte-colony stimulating factor; ICE, idarubicin + Ara-C + etoposide; MICE, mitoxantrone + etoposide + Ara-C.

**Supplementary Table 10: Pooled hazard ratios of overall survival**

|  | **Control** | **GO_ADE** | **GO_AIET** | **GO_alone** | **GO_Arac** | **GO_CCTM** | **GO_DA** | **GO_FLAG** | **GO_MICE** |
| --- | --- | --- | --- | --- | --- | --- | --- | --- | --- |
| Control | Control | 0.93 (0.68, 1.26) | 1.88 (0.49, 7.08) | 0.71 (0.51, 0.94) | 1.17 (0.7, 1.98) | 0.99 (0.47, 2.08) | 0.92 (0.77, 1.1) | 0.89 (0.59, 1.35) | 1.19 (0.8, 1.77) |
| GO_ADE | 1.08 (0.8, 1.48) | GO_ADE | 2.04 (0.52, 7.97) | 0.77 (0.49, 1.17) | 1.27 (0.7, 2.34) | 1.07 (0.48, 2.41) | 1 (0.7, 1.43) | 0.96 (0.57, 1.63) | 1.28 (0.79, 2.13) |
| GO_AIET | 0.53 (0.14, 2.03) | 0.49 (0.13, 1.93) | GO_AIET | 0.37 (0.1, 1.5) | 0.62 (0.15, 2.61) | 0.53 (0.11, 2.43) | 0.49 (0.13, 1.9) | 0.47 (0.12, 1.93) | 0.63 (0.16, 2.56) |
| GO_alone | 1.41 (1.06, 1.94) | 1.31 (0.86, 2.05) | 2.67 (0.67, 10.37) | GO_alone | 1.67 (0.93, 3.06) | 1.4 (0.64, 3.17) | 1.3 (0.93, 1.87) | 1.26 (0.77, 2.15) | 1.67 (1.05, 2.82) |
| GO_Arac | 0.85 (0.5, 1.43) | 0.79 (0.43, 1.43) | 1.61 (0.38, 6.56) | 0.6 (0.33, 1.08) | GO_Arac | 0.84 (0.34, 2.05) | 0.79 (0.45, 1.36) | 0.76 (0.39, 1.46) | 1.01 (0.53, 1.93) |
| GO_CCTM | 1.01 (0.48, 2.11) | 0.94 (0.41, 2.07) | 1.9 (0.41, 8.71) | 0.71 (0.32, 1.57) | 1.19 (0.49, 2.92) | GO_CCTM | 0.94 (0.43, 1.99) | 0.9 (0.38, 2.11) | 1.2 (0.52, 2.79) |
| GO_DA | 1.08 (0.91, 1.29) | 1 (0.7, 1.43) | 2.04 (0.53, 7.67) | 0.77 (0.53, 1.07) | 1.27 (0.74, 2.2) | 1.07 (0.5, 2.3) | GO_DA | 0.97 (0.61, 1.52) | 1.29 (0.84, 1.99) |
| GO_FLAG | 1.12 (0.74, 1.71) | 1.04 (0.61, 1.75) | 2.12 (0.52, 8.4) | 0.8 (0.47, 1.3) | 1.32 (0.69, 2.57) | 1.11 (0.47, 2.62) | 1.04 (0.66, 1.63) | GO_FLAG | 1.34 (0.75, 2.4) |
| GO_MICE | 0.84 (0.57, 1.25) | 0.78 (0.47, 1.27) | 1.59 (0.39, 6.26) | 0.6 (0.35, 0.96) | 0.99 (0.52, 1.89) | 0.83 (0.36, 1.92) | 0.78 (0.5, 1.19) | 0.75 (0.42, 1.34) | GO_MICE |

Abbreviations: GO, gemtuzumab ozogamicin; Arac, cytosine arabinoside (cytarabine); ADE, daunorubicin + Ara-C + etoposide; AIET, autologous immune enhancement therapy; CCTM, cyclophosphamide + cytarabine + topotecan + mesna; DA, daunorubicin + Ara-C; FLAG, fludarabine + Ara-C + granulocyte-colony stimulating factor; MICE, mitoxantrone + etoposide + Ara-C.

**Supplementary Table 11: Pooled hazard ratios of relapse-free survival**

|  | **Control** | **GO_ADE** | **GO_AIET** | **GO_alone** | **GO_Arac** | **GO_DA** | **GO_FLAG** | **GO_MICE** |
| --- | --- | --- | --- | --- | --- | --- | --- | --- |
| Control | Control | 0.8 (0.44, 1.44) | 1.04 (0.48, 2.24) | 0.84 (0.54, 1.23) | 0.76 (0.41, 1.4) | 0.84 (0.63, 1.09) | 0.86 (0.47, 1.59) | 1.08 (0.58, 2.03) |
| GO_ADE | 1.25 (0.69, 2.28) | GO_ADE | 1.29 (0.5, 3.41) | 1.06 (0.49, 2.09) | 0.95 (0.4, 2.22) | 1.04 (0.54, 1.99) | 1.07 (0.45, 2.53) | 1.35 (0.56, 3.18) |
| GO_AIET | 0.97 (0.45, 2.07) | 0.77 (0.29, 2.02) | GO_AIET | 0.81 (0.33, 1.9) | 0.73 (0.28, 1.93) | 0.8 (0.36, 1.78) | 0.83 (0.31, 2.22) | 1.04 (0.39, 2.75) |
| GO_alone | 1.18 (0.82, 1.85) | 0.95 (0.48, 2.03) | 1.23 (0.53, 3.03) | GO_alone | 0.9 (0.45, 1.96) | 0.99 (0.62, 1.67) | 1.02 (0.51, 2.24) | 1.27 (0.63, 2.82) |
| GO_Arac | 1.32 (0.72, 2.42) | 1.05 (0.45, 2.47) | 1.37 (0.52, 3.63) | 1.11 (0.51, 2.23) | GO_Arac | 1.1 (0.55, 2.11) | 1.13 (0.48, 2.73) | 1.42 (0.6, 3.36) |
| GO_DA | 1.2 (0.92, 1.59) | 0.96 (0.5, 1.84) | 1.24 (0.56, 2.8) | 1.01 (0.6, 1.6) | 0.91 (0.47, 1.8) | GO_DA | 1.03 (0.53, 2.03) | 1.29 (0.66, 2.58) |
| GO_FLAG | 1.16 (0.63, 2.14) | 0.93 (0.39, 2.23) | 1.21 (0.45, 3.2) | 0.98 (0.45, 1.96) | 0.89 (0.37, 2.08) | 0.97 (0.49, 1.88) | GO_FLAG | 1.26 (0.52, 3) |
| GO_MICE | 0.93 (0.49, 1.72) | 0.74 (0.31, 1.77) | 0.96 (0.36, 2.55) | 0.79 (0.35, 1.58) | 0.7 (0.3, 1.67) | 0.77 (0.39, 1.51) | 0.8 (0.33, 1.93) | GO_MICE |

Abbreviations: GO, gemtuzumab ozogamicin; Arac, cytosine arabinoside (cytarabine); ADE, daunorubicin + Ara-C + etoposide; AIET, autologous immune enhancement therapy; DA, daunorubicin + Ara-C; FLAG, fludarabine + Ara-C + granulocyte-colony stimulating factor; MICE, mitoxantrone + etoposide + Ara-C.

**Supplementary Table 12: Pooled hazard ratios of cumulative incidence of relapse**

|  | **Control** | **GO_ADE** | **GO_AIET** | **GO_alone** | **GO_DA** | **GO_FLAG** | **GO_ICE_ATRA** | **GO_MICE** |
| --- | --- | --- | --- | --- | --- | --- | --- | --- |
| Control | Control | 0.85 (0.64, 1.22) | 0.84 (0.43, 1.63) | 0.96 (0.6, 1.53) | 0.83 (0.68, 1.02) | 0.81 (0.51, 1.3) | 0.66 (0.42, 1.04) | 1.04 (0.65, 1.68) |
| GO_ADE | 1.18 (0.82, 1.55) | GO_ADE | 0.99 (0.46, 2.01) | 1.13 (0.61, 1.9) | 0.97 (0.64, 1.36) | 0.95 (0.52, 1.6) | 0.78 (0.43, 1.29) | 1.23 (0.66, 2.1) |
| GO_AIET | 1.19 (0.61, 2.34) | 1.01 (0.5, 2.2) | GO_AIET | 1.14 (0.51, 2.6) | 0.98 (0.49, 1.99) | 0.96 (0.43, 2.18) | 0.78 (0.35, 1.74) | 1.24 (0.55, 2.79) |
| GO_alone | 1.04 (0.65, 1.66) | 0.89 (0.52, 1.63) | 0.88 (0.38, 1.96) | GO_alone | 0.86 (0.52, 1.44) | 0.84 (0.44, 1.64) | 0.69 (0.36, 1.31) | 1.09 (0.56, 2.11) |
| GO_DA | 1.21 (0.98, 1.47) | 1.03 (0.74, 1.55) | 1.02 (0.5, 2.04) | 1.16 (0.7, 1.91) | GO_DA | 0.98 (0.58, 1.63) | 0.8 (0.48, 1.3) | 1.26 (0.75, 2.1) |
| GO_FLAG | 1.24 (0.77, 1.97) | 1.05 (0.62, 1.93) | 1.04 (0.46, 2.35) | 1.19 (0.61, 2.29) | 1.02 (0.61, 1.71) | GO_FLAG | 0.82 (0.42, 1.55) | 1.29 (0.67, 2.52) |
| GO_ICE_ATRA | 1.51 (0.96, 2.4) | 1.29 (0.77, 2.35) | 1.28 (0.57, 2.87) | 1.45 (0.76, 2.78) | 1.25 (0.77, 2.09) | 1.22 (0.64, 2.36) | GO_ICE_ATRA | 1.58 (0.82, 3.08) |
| GO_MICE | 0.96 (0.6, 1.54) | 0.81 (0.48, 1.51) | 0.8 (0.36, 1.83) | 0.92 (0.47, 1.77) | 0.79 (0.48, 1.34) | 0.78 (0.4, 1.5) | 0.63 (0.32, 1.22) | GO_MICE |

Abbreviations: GO, gemtuzumab ozogamicin; Arac, cytosine arabinoside (cytarabine); ADE, daunorubicin + Ara-C + etoposide; AIET, autologous immune enhancement therapy; DA, daunorubicin + Ara-C; FLAG, fludarabine + Ara-C + granulocyte-colony stimulating factor; ICE, idarubicin + Ara-C + etoposide; MICE, mitoxantrone + etoposide + Ara-C.

**Supplementary Table 13: Pooled toxicity**

| **Study** | **Year** | **Early death (30day)** | | | | | | **Oral mucositis** | | | | | |
| --- | --- | --- | --- | --- | --- | --- | --- | --- | --- | --- | --- | --- | --- |
|  |  | **GO** | **Control** | **RR** | **Lower 95% CI** | **Upper 95% CI** | **P-value** | **GO** | **Control** | **RR** | **Lower 95% CI** | **Upper 95% CI** | **P-value** |
| Loewenberg | 2010 | 7/113 | 2/119 | 3.686 | 0.782 | 17.370 | 0.099 |  |  |  |  |  |  |
| Brunnberg | 2011 | 11/57 | 3/58 | 3.731 | 1.220 | 11.414 | 0.021 |  |  |  |  |  |  |
| Delaunay | 2011 | 12/119 | 5/119 | 2.400 | 0.873 | 6.602 | 0.090 |  |  |  |  |  |  |
| Burnett | 2011 | 61/556 | 56/557 | 1.091 | 0.774 | 1.538 | 0.618 |  |  |  |  |  |  |
| Burnett | 2012 | 50/559 | 44/556 | 1.130 | 0.767 | 1.666 | 0.536 | 17/559 | 11/556 | 1.537 | 1.139 | 2.075 | 0.005 |
| Clavio | 2013 | 5/85 | 10/104 | 0.612 | 0.217 | 1.721 | 0.352 |  |  |  |  |  |  |
| Amadori | 2013 | 40/236 | 28/236 | 1.429 | 0.913 | 2.236 | 0.119 | 4/230 | 12/230 | 0.333 | 0.109 | 1.018 | 0.054 |
| Gamis | 2014 | 9/511 | 9/511 | 1.000 | 0.400 | 2.499 | 1.000 |  |  |  |  |  |  |
| Castaigne | 2014 | 5/131 | 3/137 | 1.743 | 0.425 | 7.148 | 0.440 |  |  |  |  |  |  |
| Chiara | 2016 | 4/139 | 22/270 | 0.353 | 0.124 | 1.005 | 0.051 |  |  |  |  |  |  |
| Amadori | 2016 | 8/111 | - |  |  |  |  |  |  |  |  |  |  |
| Wattad | 2017 | 2/140 | 12/247 | 0.294 | 0.067 | 1.295 | 0.106 |  |  |  |  |  |  |
| Schlenk | 2019 | 30/292 | 17/296 | 1.789 | 1.009 | 3.172 | 0.047 |  |  |  |  |  |  |
| Summary in random-effects model |  | 244/3049, 8.00% | 211/3210, 6.57% | 1.230 | 0.910 | 1.670 | 0.181 | 21/789, 2.66% | 23/786, 2.93% | 0.790 | 0.180 | 3.490 | 0.755 |
| Heterogeneity, I^2 |  |  |  | 48.50% |  |  |  |  |  | 85.10% |  |  |  |
| Heterogeneity, P-value |  |  |  | 0.030 |  |  |  |  |  | 0.01 |  |  |  |
| **Study** | **Year** | **Cardiac-related adverse effects** | | | | | | **Nausea/vomiting** | | | | | |
|  |  | **GO** | **Control** | **RR** | **Lower 95% CI** | **Upper 95% CI** | **P-value** | **GO** | **Control** | **RR** | **Lower 95% CI** | **Upper 95% CI** | **P-value** |
| Burnett | 2012 | 39/559 | 39/556 | 0.995 | 0.975 | 1.015 | 0.600 | 50/559 | 22/556 | 2.261 | 1.348 | 3.792 | 0.002 |
| Clavio | 2013 | 2/85 | 1/104 | 2.447 | 0.226 | 26.527 | 0.462 |  |  |  |  |  |  |
| Amadori | 2013 | 23/230 | 17/230 | 1.353 | 0.743 | 2.464 | 0.323 | 3/230 | 12/230 | 0.250 | 0.072 | 0.874 | 0.030 |
| Tarlock | 2016 | 11/112 | 7/71 | 0.996 | 0.405 | 2.450 | 0.993 |  |  |  |  |  |  |
| Amadori | 2016 | 31/111 | 37/114 | 0.861 | 0.577 | 1.283 | 0.461 |  |  |  |  |  |  |
| Schlenk | 2019 | 18/274 | 18/275 | 1.003 | 0.534 | 1.887 | 0.991 |  |  |  |  |  |  |
| Ho | 2019 | 2/137 | 9/548 | 0.889 | 0.194 | 4.067 | 0.879 |  |  |  |  |  |  |
| Summary in random-effects model |  | 126/1508, 8.36% | 128/1898, 6.74% | 1.000 | 0.980 | 1.010 | 1.000 | 53/789, 6.72% | 34/786, 4.33% | 0.810 | 0.090 | 6.990 | 0.849 |
| Heterogeneity, I^2 |  |  |  | 0% |  |  |  |  |  | 90.20% |  |  |  |
| Heterogeneity, P-value |  |  |  | 0.948 |  |  |  |  |  | 0.001 |  |  |  |
| **Study** | **Year** | **Hepatic-related adverse effects** | | | | | | **Gastrointestinal system** | | | | | |
|  |  | **GO** | **Control** | **RR** | **Lower 95% CI** | **Upper 95% CI** | **P-value** | **GO** | **Control** | **RR** | **Lower 95% CI** | **Upper 95% CI** | **P-value** |
| Loewenberg | 2010 | 19/113 |  |  |  |  |  | 21/113 |  |  |  |  |  |
| Delaunay | 2011 | 27/119 | 15/119 | 1.800 | 1.055 | 3.071 | 0.031 |  |  |  |  |  |  |
| Burnett | 2012 |  |  |  |  |  |  | 56/559 | 44/556 | 1.266 | 0.638 | 2.512 | 0.500 |
| Clavio | 2013 | 34/230 | 23/230 | 1.478 | 0.900 | 2.429 | 0.123 | 12/230 | 42/230 | 0.286 | 0.155 | 0.529 | 0.000 |
| Amadori | 2013 | 1/292 | 0/294 | 3.021 | 0.124 | 73.843 | 0.498 |  |  |  |  |  |  |
| Petersdorf | 2013 | 1/20 | 0/21 | 3.143 | 0.136 | 72.922 | 0.475 |  |  |  |  |  |  |
| Tarlock | 2016 |  |  |  |  |  |  | 29/112 | 26/71 | 0.707 | 0.048 | 10.327 | 0.800 |
| Amadori | 2016 | 57/111 | 52/114 | 1.126 | 0.859 | 1.475 | 0.390 |  |  |  |  |  |  |
| Schlenk | 2019 |  |  |  |  |  |  | 98/274 | 82/275 | 1.200 | 0.936 | 1.537 | 0.150 |
| Ho | 2019 | 1/137 | 3/548 | 1.333 | 0.140 | 12.719 | 0.803 |  |  |  |  |  |  |
| Summary in random-effects model |  | **140/1022, 13.70%** | **93/1326, 7.01%** | **1.290** | **1.040** | **1.600** | **0.020** | 216/1288, 16.77% | 194/1132, 17.14% | 0.770 | 0.340 | 1.740 | 0.530 |
| Heterogeneity, I^2 |  |  |  | **0%** |  |  |  |  |  | 83.80% |  |  |  |
| Heterogeneity, P-value |  |  |  | **0.648** |  |  |  |  |  | 0.000 |  |  |  |
| **Study** | **Year** | **VOD/SOS** | | | | | | **Infection** | | | | | |
|  |  | **GO** | **Control** | **RR** | **Lower 95% CI** | **Upper 95% CI** | **P-value** | **GO** | **Control** | **RR** | **Lower 95% CI** | **Upper 95% CI** | **P-value** |
| Martin | 2009 |  |  |  |  |  |  | 9/33 | 2/27 | 3.682 | 0.096 | 141.675 | 0.484 |
| Brunnberg | 2011 | 5/57 | 0/58 | 11.190 | 0.633 | 197.804 | 0.099 |  |  |  |  |  |  |
| Delaunay | 2011 | 4/127 | 0/127 | 9.000 | 0.490 | 165.451 | 0.139 |  |  |  |  |  |  |
| Clavio | 2013 |  |  |  |  |  |  | 26/85 | 25/104 | 1.273 | 0.797 | 2.032 | 0.313 |
| Amadori | 2013 |  |  |  |  |  |  | 84/230 | 127/230 | 0.661 | 0.538 | 0.813 | 0.000 |
| Petersdorf | 2013 |  |  |  |  |  |  | 5/292 | 2/294 | 2.517 | 0.492 | 12.870 | 0.268 |
| Gamis | 2014 | 18/511 | 14/511 | 1.286 | 0.646 | 2.557 | 0.474 | 182/511 | 178/511 | 1.023 | 0.866 | 1.208 | 0.793 |
| Castaigne | 2014 | 6/131 | 2/137 | 3.137 | 0.645 | 15.267 | 0.157 | 102/131 | 106/137 | 1.006 | 0.885 | 1.144 | 0.923 |
| Tarlock | 2016 | 6/112 | 2/71 | 1.902 | 0.395 | 9.164 | 0.423 | 65/112 | 41/71 | 1.005 | 0.780 | 1.295 | 0.969 |
| Amadori | 2016 |  |  |  |  |  |  | 49/111 | 48/114 | 1.048 | 0.777 | 1.416 | 0.758 |
| Schlenk | 2019 |  |  |  |  |  |  | 174/274 | 161/275 | 1.085 | 0.942 | 1.250 | 0.260 |
| Ho | 2019 | 6/137 | 22/548 | 1.100 | 0.430 | 2.810 | 0.850 | 9/137 | 46/548 | 0.783 | 0.393 | 1.559 | 0.486 |
| Summary in random-effects model |  | **45/1075, 4.19%** | **40/1452, 2.75%** | **1.560** | **0.960** | **2.530** | **0.072** | 705/1916, 36.80% | 736/2311, 31.85% | 0.980 | 0.860 | 1.110 | 0.756 |
| Heterogeneity, I^2 |  |  |  | **0%** |  |  |  |  |  | 58.90% |  |  |  |
| Heterogeneity, P-value |  |  |  | **0.435** |  |  |  |  |  | 0.013 |  |  |  |
| **Study** | **Year** | **Pulmonary/upper respiratory** | | | | | | **Bleeding** | | | | | |
|  |  | **GO** | **Control** | **RR** | **Lower 95% CI** | **Upper 95% CI** | **P-value** | **GO** | **Control** | **RR** | **Lower 95% CI** | **Upper 95% CI** | **P-value** |
| Amadori | 2013 |  |  |  |  |  |  | 24/230 | 23/230 | 1.044 | 0.607 | 1.794 | 0.878 |
| Petersdorf | 2013 | 3/292 | 0/294 | 7.048 | 0.366 | 135.841 | 0.196 |  |  |  |  |  |  |
| Castaigne | 2014 |  |  |  |  |  |  | 118/131 | 107/137 | 1.153 | 1.038 | 1.281 | 0.008 |
| Tarlock | 2016 | 10/112 | 9/71 | 0.704 | 0.301 | 1.648 | 0.419 | 5/112 | 1/71 | 3.170 | 0.216 | 46.533 | 0.400 |
| Amadori | 2016 |  |  |  |  |  |  | 28/111 | 34/114 | 0.846 | 0.552 | 1.296 | 0.442 |
| Schlenk | 2019 | 22/274 | 22/275 | 1.004 | 0.569 | 1.769 | 0.990 | 22/274 | 21/275 | 1.051 | 0.548 | 2.015 | 0.880 |
| Ho | 2019 | 3/137 | 13/548 | 0.923 | 0.267 | 3.194 | 0.899 |  |  |  |  |  |  |
| Summary in random-effects model |  | 38/815, 4.66% | 44/1188, 3.70% | 0.940 | 0.610 | 1.460 | 0.781 | **197/858, 22.96%** | **186/827, 22.49%** | **1.130** | **1.020** | **1.250** | **0.018** |
| Heterogeneity, I^2 |  |  |  | 0% |  |  |  |  |  | **0%** |  |  |  |
| Heterogeneity, P-value |  |  |  | 0.517 |  |  |  |  |  | **0.626** |  |  |  |
| **Study** | **Year** | **Constitutional symptoms** | | | | | | **Metabolic/laboratory adverse effects** | | | | | |
|  |  | **GO** | **Control** | **RR** | **Lower 95% CI** | **Upper 95% CI** | **P-value** | **GO** | **Control** | **RR** | **Lower 95% CI** | **Upper 95% CI** | **P-value** |
| Tarlock | 2016 | 8/112 | 2/71 | 2.536 | 0.554 | 11.602 | 0.230 | 34/112 | 26/71 | 0.829 | 0.547 | 1.256 | 0.376 |
| Amadori | 2016 |  |  |  |  |  |  | 18/111 | 17/114 | 1.087 | 0.591 | 2.000 | 0.787 |
| Schlenk | 2019 | 34/274 | 26/275 | 1.313 | 0.801 | 2.150 | 0.280 | 83/274 | 88/275 | 0.947 | 0.709 | 1.264 | 0.710 |
| Summary in random-effects model |  | 42/386, 10.88% | 28/346, 8.09% | 1.400 | 0.870 | 2.240 | 0.163 | 135/497, 27.16% | 131/460, 28.48% | 0.930 | 0.740 | 1.160 | 0.527 |
| Heterogeneity, I^2 |  |  |  | 0% |  |  |  |  |  | 0% |  |  |  |
| Heterogeneity, P-value |  |  |  | 0.420 |  |  |  |  |  | 0.756 |  |  |  |
| **Study** | **Year** | **Pain** | | | | | | **Skin or mucosa** | | | | | |
|  |  | **GO** | **Control** | **RR** | **Lower 95% CI** | **Upper 95% CI** | **P-value** | **GO** | **Control** | **RR** | **Lower 95% CI** | **Upper 95% CI** | **P-value** |
| Tarlock | 2016 | 19/112 | 13/71 | 0.927 | 0.489 | 1.757 | 0.815 | 9/112 | 4/71 | 1.426 | 0.456 | 4.458 | 0.541 |
| Schlenk | 2019 | 28/274 | 26/275 | 1.081 | 0.626 | 1.866 | 0.780 | 12/274 | 20/275 | 0.602 | 0.300 | 1.208 | 0.153 |
| Summary in random-effects model |  | 47/386, 12.18% | 39/346, 11.27% | 1.010 | 0.670 | 1.530 | 0.962 | 21/386, 5.44% | 24/346, 6.94% | 0.820 | 0.360 | 1.840 | 0.633 |
| Heterogeneity, I^2 |  |  |  | 0% |  |  |  |  |  | 37.50% |  |  |  |
| Heterogeneity, P-value |  |  |  | 0.720 |  |  |  |  |  | 0.206 |  |  |  |
| **Study** | **Year** | **Renal/genitourinary** | | | | | | **Neurology** | | | | | |
|  |  | **GO** | **Control** | **RR** | **Lower 95% CI** | **Upper 95% CI** | **P-value** | **GO** | **Control** | **RR** | **Lower 95% CI** | **Upper 95% CI** | **P-value** |
| Petersdorf | 2013 |  |  |  |  |  |  | 4/292 | 1/294 | 4.027 | 0.453 | 35.818 | 0.212 |
| Tarlock | 2016 | 3/112 | 1/71 | 1.902 | 0.202 | 17.928 | 0.574 | 4/112 | 1/71 | 2.536 | 0.289 | 22.232 | 0.401 |
| Amadori | 2016 | 7/111 | 9/114 | 0.799 | 0.308 | 2.071 | 0.644 |  |  |  |  |  |  |
| Schlenk | 2019 | 14/274 | 15/275 | 0.937 | 0.461 | 1.903 | 0.857 | 9/274 | 8/275 | 1.129 | 0.442 | 2.884 | 0.800 |
| Ho | 2019 | 0/137 | 1/548 | 1.326 | 0.054 | 32.375 | 0.863 | 0/137 | 1/548 | 1.326 | 0.054 | 32.375 | 0.863 |
| Summary in random-effects model |  | 24/634, 3.79% | 26/1008, 2.58% | 0.940 | 0.540 | 1.610 | 0.824 | 17/815, 2.09% | 11/1188, 0.93% | 1.480 | 0.680 | 3.230 | 0.324 |
| Heterogeneity, I^2 |  |  |  | 0% |  |  |  |  |  | 0% |  |  |  |
| Heterogeneity, P-value |  |  |  | 0.911 |  |  |  |  |  | 0.713 |  |  |  |
| **Study** | **Year** | **Allergy/immunology** | | | | | | **Coagulation** | | | | | |
|  |  | **GO** | **Control** | **RR** | **Lower 95% CI** | **Upper 95% CI** | **P-value** | **GO** | **Control** | **RR** | **Lower 95% CI** | **Upper 95% CI** | **P-value** |
| Tarlock | 2016 |  |  |  |  |  |  | 7/112 | 2/71 | 2.219 | 0.474 | 10.382 | 0.311 |
| Schlenk | 2019 | 9/274 | 4/275 | 2.258 | 0.704 | 7.246 | 0.171 | 2/274 | 4/275 | 0.502 | 0.093 | 2.717 | 0.424 |
| Summary in random-effects model |  | 9/274, 3.28% | 4/275, 1.45% | 2.258 | 0.704 | 7.246 | 0.171 | 9/386, 2.33% | 6/346, 1.73% | 1.100 | 0.260 | 4.710 | 0.897 |
| Heterogeneity, I^2 |  |  |  | - |  |  |  |  |  | 38.40% |  |  |  |
| Heterogeneity, P-value |  |  |  | - |  |  |  |  |  | 0.203 |  |  |  |
| **Study** | **Year** | **Vascular** | | | | | | **Ocular/visual** | | | | | |
|  |  | **GO** | **Control** | **RR** | **Lower 95% CI** | **Upper 95% CI** | **P-value** | **GO** | **Control** | **RR** | **Lower 95% CI** | **Upper 95% CI** | **P-value** |
| Tarlock | 2016 |  |  |  |  |  |  | 1/112 | 1/71 | 0.634 | 0.040 | 9.974 | 0.746 |
| Schlenk | 2019 | 1/274 | 4/275 | 0.251 | 0.028 | 2.231 | 0.215 | 1/274 | 1/275 | 1.004 | 0.063 | 15.965 | 0.998 |
| Summary in random-effects model |  | 1/274, 0.36% | 4/275, 1.45% | 0.251 | 0.028 | 2.231 | 0.215 | 2/386, 0.52% | 2/346, 0.58% | 0.800 | 0.110 | 5.630 | 0.824 |
| Heterogeneity, I^2 |  |  |  | - |  |  |  |  |  | 0% |  |  |  |
| Heterogeneity, P-value |  |  |  | - |  |  |  |  |  | 0.818 |  |  |  |
| **Study** | **Year** | **Musculoskeletal/soft tissue** | | | | | | **Neutropenic fever** | | | | | |
|  |  | **GO** | **Control** | **RR** | **Lower 95% CI** | **Upper 95% CI** | **P-value** | **GO** | **Control** | **RR** | **Lower 95% CI** | **Upper 95% CI** | **P-value** |
| Amadori | 2013 |  |  |  |  |  |  | 59/230 | 71/230 | 0.831 | 0.620 | 1.114 | 0.215 |
| Gamis | 2014 |  |  |  |  |  |  | 163/511 | 158/511 | 1.032 | 0.861 | 1.237 | 0.736 |
| Tarlock | 2016 | 2/112 | 0/71 | 3.186 | 0.155 | 65.409 | 0.452 |  |  |  |  |  |  |
| Amadori | 2016 |  |  |  |  |  |  | 20/111 | 27/114 | 0.761 | 0.454 | 1.274 | 0.299 |
| Summary in random-effects model |  | 2/112, 1.79% | 0/71, 0% | 3.186 | 0.155 | 65.409 | 0.452 | 242/852, 28.40% | 256/855, 29.94% | 0.940 | 0.790 | 1.110 | 0.476 |
| Heterogeneity, I^2 |  |  |  | - |  |  |  |  |  | 13.50% |  |  |  |
| Heterogeneity, P-value |  |  |  | - |  |  |  |  |  | 0.315 |  |  |  |

Abbreviations: GO, gemtuzumab ozogamicin; RR, relative risk; 95% CI, confidence interval; VOD/SOD, Hepatic veno-occlusive disease (VOD) or sinusoidal obstruction syndrome.

**Supplementary Table 14: Publication bias based on Egger’s and Begg’s tests**

| **Endpoints** | **Total number of studies** | **P-value of Egger's test** | **P-value of Begg's test** |
| --- | --- | --- | --- |
| CR | 18 | 0.030 | 0.112 |
| OS | 23 | 0.053 | 0.091 |
| EFS | 11 | 0.777 | 1.000 |
| RFS | 18 | **0.017** | **0.049** |
| CIR | 14 | 0.995 | 1.000 |

Abbreviations: CR, complete remission; OS, overall survival; EFS, event-free survival; RFS, relapse-free survival; CIR, cumulative incidence of relapse. P-value in bold meant the statistically significant.

## Supplementary Figures


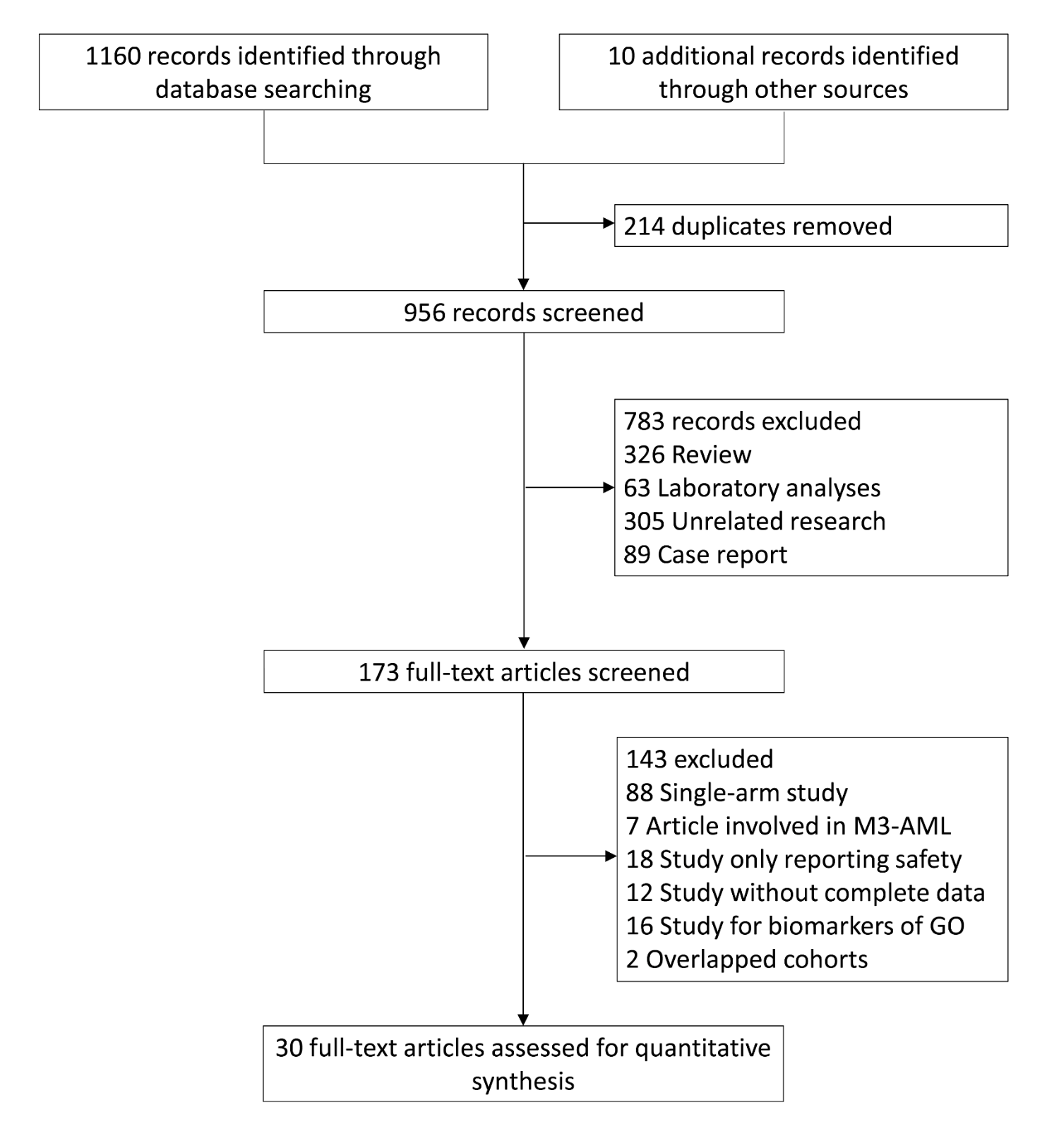


**Supplementary Figure 1.** **Flow diagram of the study selection.** Abbreviations: AML, acute myeloid leukemia; GO, gemtuzumab ozogamicin.


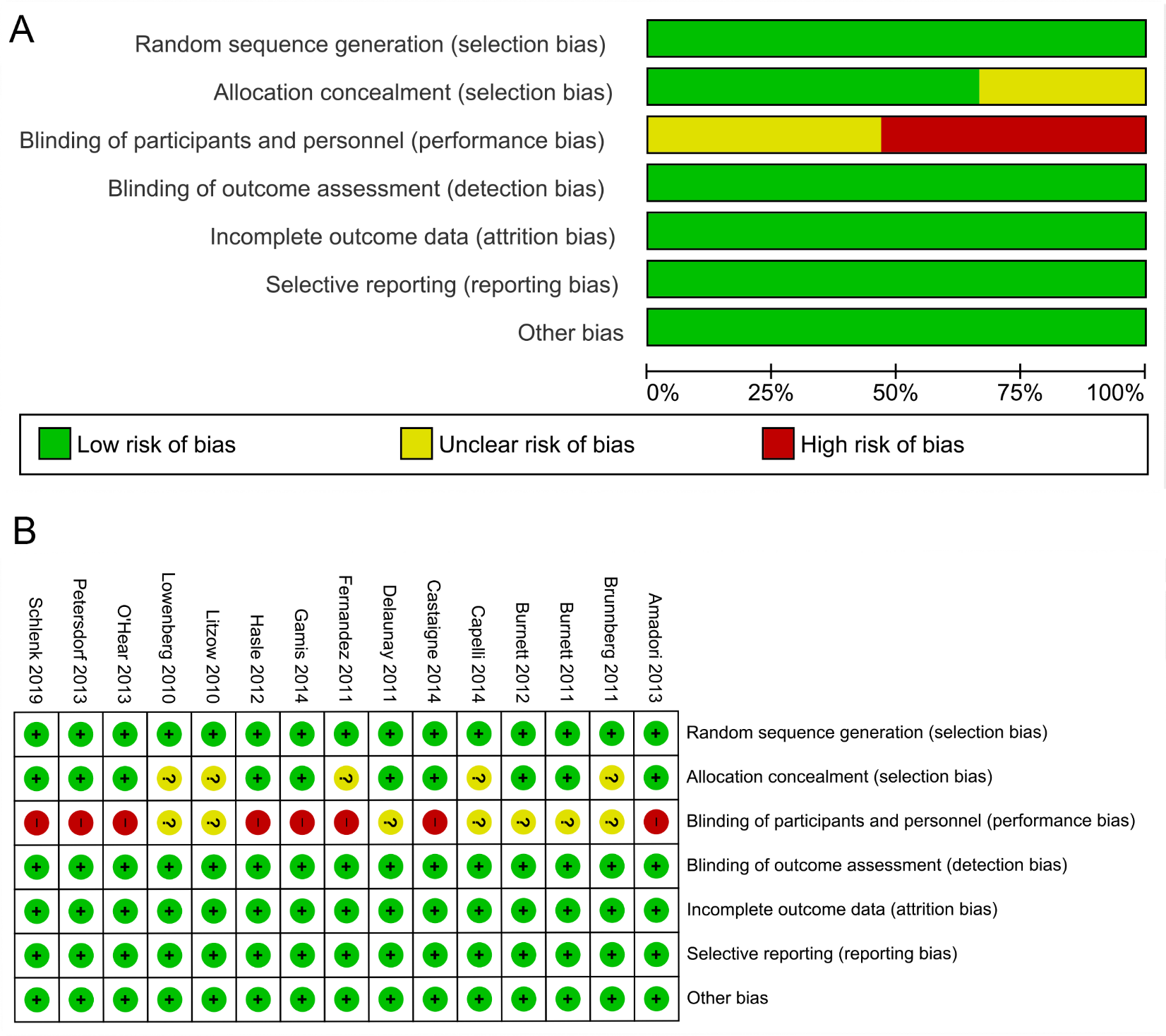


**Supplementary Figure 2.** **Quality assessment of randomized controlled trials.** (A) Risk of bias graph in quality assessment for randomized controlled trials. (B) Risk of bias summary in quality assessment for randomized controlled trials.


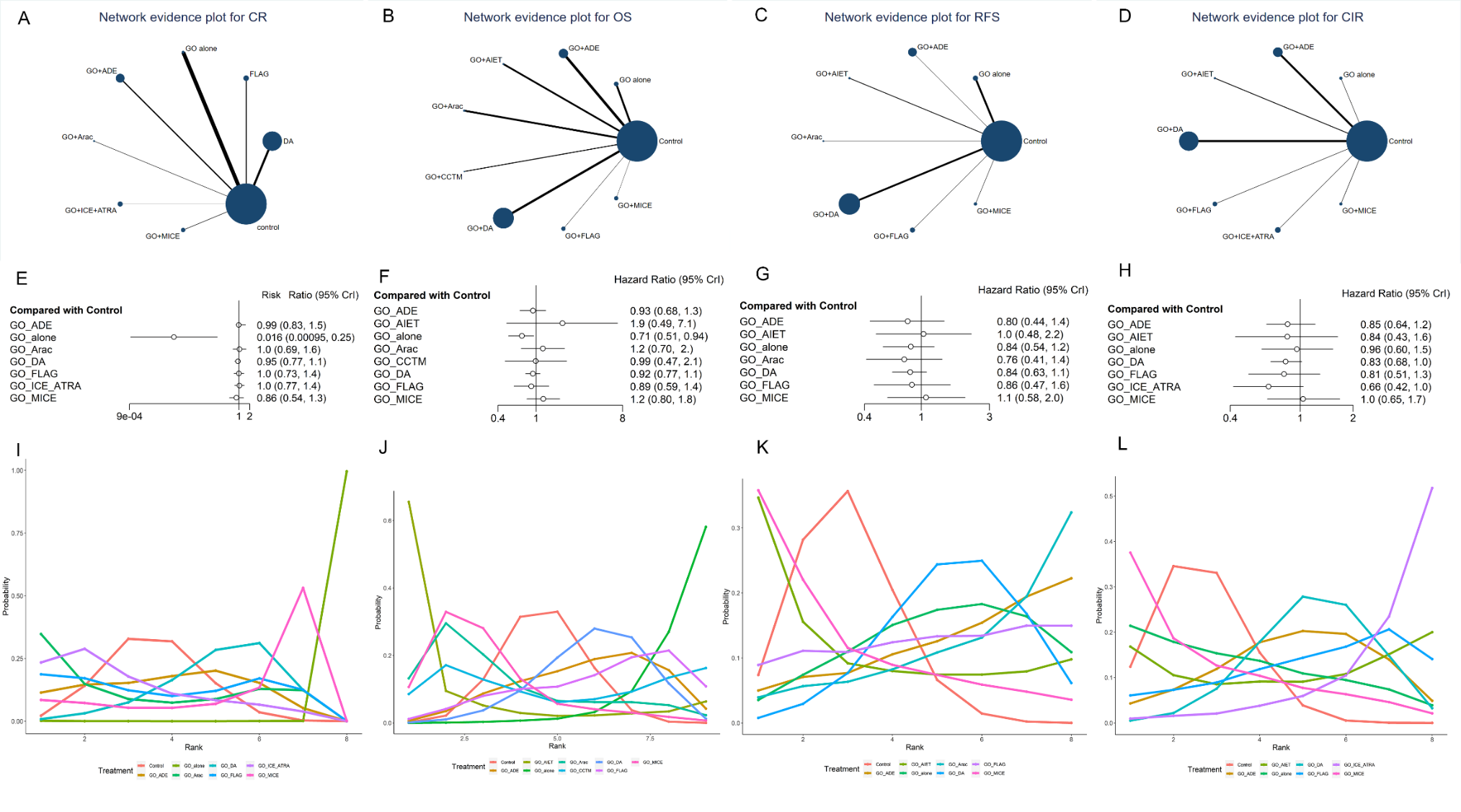


**Supplementary Figure 3. Network of analyzed comparison in RCT.** Network evidence plot for CR (A), OS (B), RFS (C), and CIR (D). (E) Comparison of RR for CR between GO-related treatment and control. Comparison of HR for OS (F), RFS (G), and CIR (H) between GO-related treatment and control. The rank of probability for improved CR (I), OS (J), RFS (K), and CIR (L) among GO and non-GO groups. RR, risk ratio; HR, hazard ratio; RCT, randomized controlled trial; CR, complete remission; OS, overall survival; RFS, relapse-free survival; CIR, cumulative incidence of relapse; GO, gemtuzumab ozogamicin; Ara-C, cytosine arabinoside (cytarabine); DA, daunorubicin + Ara-C; FLAG, fludarabine + Ara-C + granulocyte-colony stimulating factor; ADE, daunorubicin + Ara-C + etoposide; MICE, mitoxantrone + etoposide + Ara-C; ICE, idarubicin + Ara-C + etoposide; AIET, autologous immune enhancement therapy; CCTM, cyclophosphamide + cytarabine + topotecan + mesna.


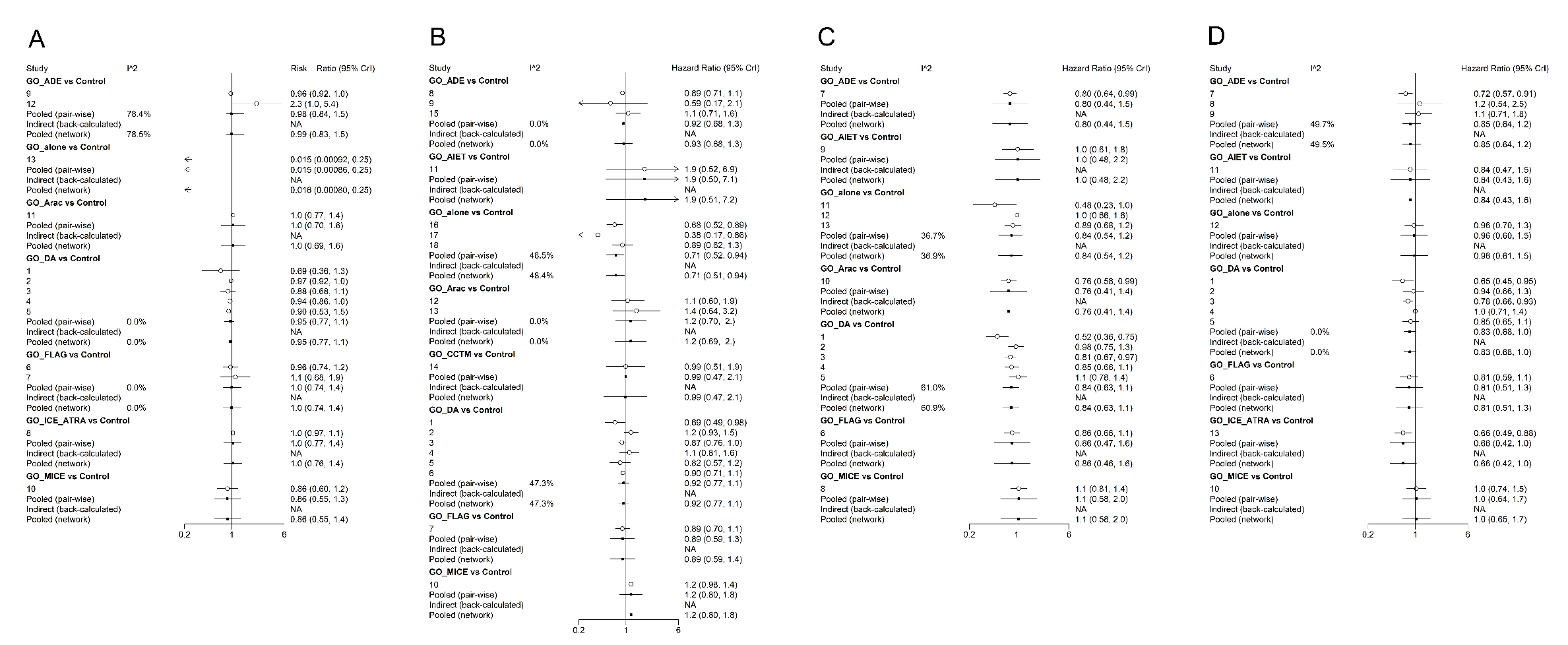


**Supplementary Figure 4. Analyses of heterogeneity in endpoints.** (A) Complete remission. (B) Overall survival. (C) Relapse-free survival. (D) Cumulative incidence of relapse. GO, gemtuzumab ozogamicin; Ara-C, cytosine arabinoside (cytarabine); DA, daunorubicin + Ara-C; FLAG, fludarabine + Ara-C + granulocyte-colony stimulating factor; ADE, daunorubicin + Ara-C + etoposide; MICE, mitoxantrone + etoposide + Ara-C; ICE, idarubicin + Ara-C + etoposide; AIET, autologous immune enhancement therapy; CCTM, cyclophosphamide + cytarabine + topotecan + mesna.


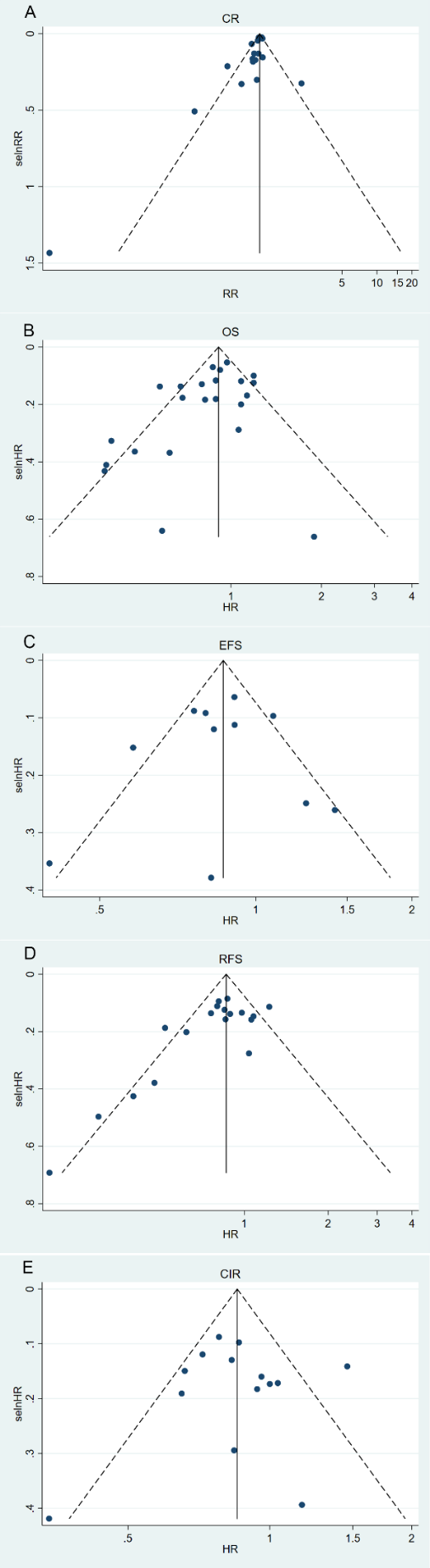


**Supplementary Figure 5. Funnel plots for endpoints of GO.** (A) Complete remission. (B) Overall survival. (C) Event-free survival. (D) Relapse-free survival. (E) Cumulative incidence of relapse. GO, gemtuzumab ozogamicin; RR, relative risk; HR, hazard ratio.
